# Enhancing Weight Loss Programs: A Meta‐Analysis of Financial Incentives and Behavior Change Techniques

**DOI:** 10.1111/obr.70124

**Published:** 2026-03-18

**Authors:** Sara M. Hondmann, Milon H. M. van Vliet, Roxy A. van Eersel, Linda D. Breeman, Mike Keesman, David R. de Buisonjé, George Haroon, Roderik Kraaijenhagen, Veronica R. Janssen, Andrea W. M. Evers

**Affiliations:** ^1^ Health, Medical and Neuropsychology Unit, Institute of Psychology Leiden University Leiden the Netherlands; ^2^ Department of Public Health and Primary Care Leiden University Medical Center Leiden the Netherlands; ^3^ National eHealth Living Lab Leiden University Medical Center Leiden the Netherlands; ^4^ Department of Human‐Centered Design, Faculty of Industrial Design Engineering Delft University of Technology Delft the Netherlands; ^5^ Department of Economics and Business Economics, Radboud school of Management Radboud University Nijmegen the Netherlands; ^6^ Hearts4People Foundation Amsterdam the Netherlands; ^7^ Department of Cardiology Leiden University Medical Center Leiden the Netherlands; ^8^ Department of Psychiatry Leiden University Medical Center Leiden the Netherlands; ^9^ Medical Delta Leiden University, Delft University of Technology, Erasmus University Delft the Netherlands

**Keywords:** behavior change techniques, financial incentives, health behavior, interventions, meta‐analysis, weight loss

## Abstract

Obesity is a risk factor for chronic illness. Financial incentives contingent on behaviors or outcomes related to weight loss can promote weight loss efforts. This meta‐analysis examines the added effect of financial incentives for weight loss in health behavior change interventions, both short‐ and long‐term, in adults. Additionally, subgroup analyses explored incentive targets (physiological outcomes, lifestyle behaviors, process behaviors, or multiple targets) and types of behavior change techniques (BCTs; interactive or passive) as moderators. Seven databases were systematically searched for (quasi‐)randomized control trial studies. The meta‐analysis included 29 studies with incentive and control conditions, 2396 adults in control and 3397 in incentive conditions. Using a random‐effects model, results showed a small, significant, positive effect of incentives on weight loss during the incentive phase (i.e., short‐term; 51 comparisons; SMD = 0.180, *p* < 0.001) but not in the post‐incentive phase (i.e., long‐term, after incentive removal; 23 comparisons; SMD = 0.032, *p* = 0.432). However, limited studies reported post‐incentive data. In the incentive phase, subgroup analyses showed that incentivizing multiple targets and physiological outcomes has a larger effect than incentivizing lifestyle and process behaviors. Subgroup analyses showed no significant effect of type of BCTs. However, in the incentive phase, the within‐group analysis showed that combining incentives with interactive BCTs had a significant effect while passive BCTs did not. Suggesting that combining incentives only with passive BCTs may not be sufficient to achieve weight loss, given its complexity. In conclusion, these findings suggest that financial incentive interventions are effective on short‐term weight loss, especially when incentivizing multiple targets or physiological outcomes.

## 1 Introduction

The global prevalence of obesity has increased significantly, with one in eight adults affected, posing a major risk for chronic illnesses like cardiovascular disease and diabetes [1, 2]. A healthy diet and regular physical activity help reduce this risk [2]. Despite intentions to adopt healthier behaviors, many individuals struggle to start and maintain them [3, 4], the so‐called intention‐behavior gap [5]. To bridge this gap [5], a better understanding of effective intervention strategies is needed. One possible approach is financial incentives [6, 7], which could motivate people to adopt and sustain health behavior change [8, 9]. Financial incentives are defined as reinforcements (e.g., money, coupons, or gifts) that motivate people to perform a behavior [10]. Previous reviews and meta‐analyses have shown that incentives are generally effective in the short term across different behaviors; however, their effectiveness varies by context, behavior, duration, and intervention design [7, 11, 12, 13, 14, 15, 16, 17, 18, 19, 20, 21, 22, 23].

Financial incentives can vary in their form (e.g., cash and vouchers) and features (e.g., incentive magnitude, target, certainty, and recipient) [6, 24]. A higher magnitude can improve outcomes [7, 22, 25]. Furthermore, immediate [14, 17, 25, 26] and frequent [6] delivery of incentives can strengthen effects [25], especially for outcomes like weight loss, which result from multiple behaviors (e.g., increased physical activity and healthy diet) and are difficult to achieve and maintain long‐term [12, 25]. These possible variations in incentive design underscore the importance of investigating what features are effective, when, and for whom [22, 24, 25, 27, 28]. While identifying an optimal incentive design is crucial, understanding whether behavior change persists after incentive removal is equally important. Incentives cannot be provided indefinitely due to limited recourses, making sustained behavior change essential. Reviews show mixed results on long‐term effect of incentives on health behavior [11, 28], with some suggesting possible long‐term effects [13, 17, 18, 29, 30], and others none [12, 14, 21, 22, 23]. Therefore, the effect of financial incentives should be examined during both the *incentive* and *post‐incentive* (i.e., after incentive termination) *phase*.

One of the variations in incentive interventions is the target (i.e., behavior or outcomes) on which an incentive is contingent. Incentives can target: (1) *lifestyle behaviors* (e.g., walking) [31], (2) *process behaviors* (e.g., gym attendance) [32], both of which are considered healthy in themselves, can be modulated directly, and can therefore be used to set proximal health goals [24, 25], (3) *physiological outcomes* (e.g., weight loss) [33], which largely depend on behavior change, and are therefore indirectly modifiable and used to set distal health goals [24, 25], or (4) a combination of *multiple targets*. A previous systematic review showed some evidence that incentivizing lifestyle and process behaviors is more effective for weight loss than incentivizing physiology outcomes such as weight loss itself; however, this conclusion was based on a small sample [21]. This study examines whether incentive target affects the effectiveness of the financial incentive.

Another variation in interventions is how financial incentives interact with other behavior change techniques (BCTs) as they are often combined [34] rather than implemented alone [3, 6]. Adding incentives to an intervention can possibly double the odds of success [19, 35]. According to the COM‐B model for behavior change [36], behavior change requires capability, opportunity, and motivation. Financial incentives enhance opportunity by reducing barriers to engage in health behavior, by for instance providing means to healthy food and physical activity facilities [36, 37], and increase motivation through immediate rewards [28]. Additionally, other BCTs (e.g., providing feedback and self‐monitoring) can increase someone's capability and motivation to perform healthy behaviors [36]. According to the Self‐Determination Theory by Deci (1999), motivation can be intrinsic or extrinsic [38]. Concerns have been raised that external rewards, such as financial incentives, may “crowd out” intrinsic motivation by shifting behavior from internal choice to external control. Although some studies support this effect [38, 39], others do not [40, 41]. To illustrate, meta‐analyses show that health behaviors can return to baseline after incentives end, but do not necessarily fall below [13, 15, 19, 30], suggesting no consistent crowding‐out effect. Some studies even report lasting effects [13, 17, 18, 29, 30], whereas others do not [12, 14, 21, 22, 23]. Such variability might also indicate that other intervention components (i.e., BCTs) may influence motivation and support maintenance. Financial incentives may help initiate change, but additional BCTs may be needed to sustain it. How incentives and BCTs jointly affect motivation, especially long‐term, remains unclear.

Following Michie et al. (2009), we distinguish between interactive and passive BCTs. *Interactive BCTs* are defined as those that require active participation from participants (e.g., setting goals, planning, coaching sessions), while *passive BCTs* only require passive information receipt (e.g., information provision through flyers and websites) [42]. We use the term “interactive” throughout this review, rather than “active” as in Michie et al. (2009), to emphasize the engagement required from participants. Previous studies suggest that interactive interventions, which actively engage participants in the behavior change process, are more effective [42, 43]. This may be because such interventions provide a more tailored approach and increase participants' engagement [44], self‐efficacy, and competence. In turn, these factors can support more effective behavioral change and better health outcomes, such as weight loss [42, 44, 45, 46]. This is further supported by previous review on physical activity showing that tailored interventions are more effective than non‐tailored [47]. This study therefore examines whether financial incentives are more effective when combined with passive or interactive BCTs.

Despite growing interest in financial incentives, a significant knowledge gap remains. As discussed, it is unclear what the short and long‐term effectiveness of incentives is. Furthermore, it is unclear how incentives vary by incentive target or by types of BCTs. To address these gaps, this meta‐analysis investigates the effectiveness of financial incentives for weight loss over time and whether this varies based on incentive targets or when combined with different types of BCTs. First, we expect an overall positive effect of incentives, leading to more weight loss than interventions without incentives [6, 7]. Second, we expect that (a) incentivizing multiple targets is more effective than solely incentivizing lifestyle behaviors [6, 25], (b) incentivizing lifestyle behaviors is more effective than incentivizing process behaviors [25], and (c) targeting physiology outcomes is least effective for weight loss [21]. Third, we expect incentive interventions to be more effective for weight loss when combined with interactive BCTs than with only passive BCTs [42, 45]. Lastly, given the mixed findings, we will exploratively investigate the effectiveness of incentives separately in the *incentive* and *post‐incentive* (i.e., after incentive removal) phases to understand the lasting effects after their termination.

## 2 Methods

### 2.1 Protocol and Registration

This study followed the Preferred Reporting Items for Systematic Reviews and Meta‐Analyses (PRISMA) guidelines [48] and was registered in the PROSPERO International prospective register of systematic reviews before data extraction commenced (2021: https://www.crd.york.ac.uk/PROSPERO/view/CRD42021239275).

### 2.2 Eligibility Criteria

Eligibility criteria, defined by the PICO framework, were as follows: (P) healthy adults or adults (mean sample age ≥ 18 years.) with (or increased risk of) chronic conditions, (I) financial incentives contingent on health behaviors or outcomes related to weight loss (e.g., physical activity and gym attendance) and the recipient of the incentive is an individual, (C) a (quasi‐)RCT design with a non‐incentivized control group, (O) outcomes were objective or self‐reported weight (in pounds, kilograms, or BMI) measured at least at baseline and the last time‐point of the incentive phase. Furthermore, the studies had to be written in English.

Since a healthy diet and regular physical activity are seen as weight loss promotion strategies [2], interventions targeting diet and/or physical activity were included if weight outcomes were reported. Exclusion criteria were as follows: (1) secondary analysis article, (2) incentives only to promote weight maintenance, (3) incentives not contingent on health behaviors or outcomes unrelated to weight loss (e.g., incentives for participation or adherence), or (4) incentives provided to groups or indirectly to the individual.

### 2.3 Literature Search

Studies were identified using a two‐step search strategy. First, the electronic databases PubMed, PsycINFO (EbscoHost), Embase (OVID), Web of Science, Emcare (OVID), COCHRANE Library (CENTRAL), and Academic Search Premier (EbscoHost) were searched with help from an experienced librarian (JS) to identify relevant studies published from database inception to April 26, 2024. The search string (see Supporting Information S1: file S1) consisted of keywords related to the study design, intervention, health behaviors, and outcomes of interest. Reference lists of relevant reviews and eligible articles found were searched to identify additional studies.

### 2.4 Screening

Duplicates were removed and a standardized, pre‐piloted screening form and manual (see File S2) was used for screening the obtained studies on the eligibility criteria. First, titles and abstracts were screened by MS (literature search 2021), GH (search update 2022), and SH (search update 2024) using the artificial intelligence (AI) screening tool ASReview [49]. The AI screening tool used active learning to sort the titles and abstracts from most to least relevant based on screening decisions (i.e., deciding to include or exclude a study) of the reviewer. After 150 abstracts were labeled as non‐eligible in a row, the title and abstract screening procedure was stopped [50, 51]. To ensure reliability and objectivity, MV (first searches) and RE (search update 2024) independently and randomly double‐screened 20% of all the titles and abstracts that resulted from the two‐step search strategy using screening software Rayyan [52]. The raw inter‐rater agreement between the screening choices of all searches was 97% (Cohen's Kappa = 0.62). The moderate‐to‐substantial agreement shows the reliability of the screening process and the AI screening tool ASReview [53, 54]. Second, full texts of potentially eligible studies were independently screened by MS/GH and MV and SH and RE using the pre‐piloted screening form and manual, and reasons for exclusion were recorded. The substantial‐to‐high inter‐rater agreement (90%, Cohen's Kappa = 0.78) showed again the reliability of the screening process [53, 54]. Disagreements were resolved by consensus or the involvement of a third reviewer, L. B. or M. V.

### 2.5 Data Extraction

A standardized, prepiloted coding form and manual were used for data extraction (see Supporting Information S1: file S2). This coding form was based on the Cochrane Data Collection form, two incentive intervention frameworks [24, 25], and the BCT taxonomy V1 [34]. The following data items were extracted: general information about the article (e.g., date of publication and main objective), sample characteristics, intervention and incentive characteristics (e.g., control group, type of incentive, incentive frequency, and magnitude), used incentive target (lifestyle behaviors, process behaviors, physiological outcome, or multiple targets) and types of BCTs (only passive versus passive and interactive) provided next to the incentives (moderators), measure of effectiveness, and risk of bias (using Cochrane Collaboration Risk of Bias Tool [55]). The total incentive magnitude reported in an article was converted into US$ using the currency exchange rate at the date of publication of the specific article, to be able to compare studies (https://www.wisselkoers.nl/calculator). The outcome that was extracted as a measure of effectiveness were objective or self‐report measurements of weight (absolute or relative weight [difference] in pounds, kilograms, and BMI) at different time points for the incentive and post‐incentive phase. When studies had to deal with missing data and reported intention‐to‐treat analyses with a missing data imputation method, the outcomes of these analyses were used.

Extraction of the data was done independently by MS/MV, GH/RE, and SH/RE. In case of missing data items, authors were contacted with a request to provide this data. Studies were excluded from the meta‐analysis when there was missing data on the outcome of interest and the authors did not respond after being contacted twice. The raw inter‐coder agreement was 85% which showed the reliability of the coding process [53]. Disagreements were again resolved by consensus or the involvement of a third reviewer (LB or MV).

### 2.6 Risk of Bias Assessment

The quality of the studies was assessed using the Cochrane Collaboration Risk of Bias Tool [55]. Studies were scored on the following risks of bias: random sequence generation, allocation concealment, blinding of participants and personnel, blinding of outcome assessment, incomplete outcome data, selective reporting, and other biases.

### 2.7 Data Analyses

The incentive phase included all outcomes before incentive removal (from baseline measurement to the last intervention assessment). The post‐incentive phase starts at the last intervention assessment till the last follow‐up measurement. When studies reported multiple time points within a phase, data from the first and final assessment within that phase were extracted. This approach enabled capturing the longest possible duration of both the incentive and post‐incentive phases while maintaining consistent criteria across studies. For studies whose outcomes are compared to baseline, a mean change was calculated for the period between last intervention assessment till the last follow‐up measurement.

Meta‐analyses, categorized by incentive and post‐incentive phase, were conducted using a random‐effects model with the software Comprehensive Meta‐Analysis (CMA) Version 3 [56]. Standardized mean differences (SMDs) between the incentive and control conditions, along with 95% confidence intervals (CI), were calculated and presented in a forest plot to assess the effect of financial incentives on short‐term (incentive phase) and long‐term weight differences (post‐incentive phase). SMDs were reported instead of raw mean differences, since included studies use different measurement units for weight differences. The reported post‐intervention or follow‐up sample sizes (not the baseline sample sizes) were used to calculate effect sizes in CMA, for this is most conservative. Effect sizes were calculated as Hedges' g in CMA, based on the pre‐post mean change score for independent groups. Furthermore, CMA uses the correlation between time‐points to calculate effect sizes, which was often not reported in the included studies. Therefore, to prevent overestimation of the true effect, again the most conservative option was chosen, which was using a correlation of zero. If multiple incentive conditions within one study utilized the same control conditions, this control group sample size was divided by the number of incentive conditions using it and entered in CMA as separate comparisons with their respective interventions. Heterogeneity between studies was assessed by examining forest plots and calculating the *I* ^2^‐ and *Q*‐statistic. For interpretation, *I* ^2^ values of 20, 45, 70, and 85 are considered low, moderate, substantial and considerable heterogeneity respectively [57]. In addition, mixed‐effects moderator analyses (subgroup analyses) were performed to assess the difference in effectiveness based on the different incentive targets (i.e., lifestyle behaviors, process behaviors, physiological outcomes, or multiple targets), and the combination of incentives with different types of BCTs (i.e., passive or interactive). Type of BCTs were defined based on previous work by Michie et al. (2009) [42]. Interactive BCTs were characterized by involving active participant engagement (e.g., group sessions, individual coaching or counseling sessions). Passive BCTs only require passive information receipt (e.g., information provision through flyers and websites). Finally, funnel plots, the trim‐and‐fill procedure, the fail‐safe number, and the rank correlation test (i.e., Kendall's Tau) were used to assess the presence of publication bias [58].

## 3 Results

**FIGURE 1:**
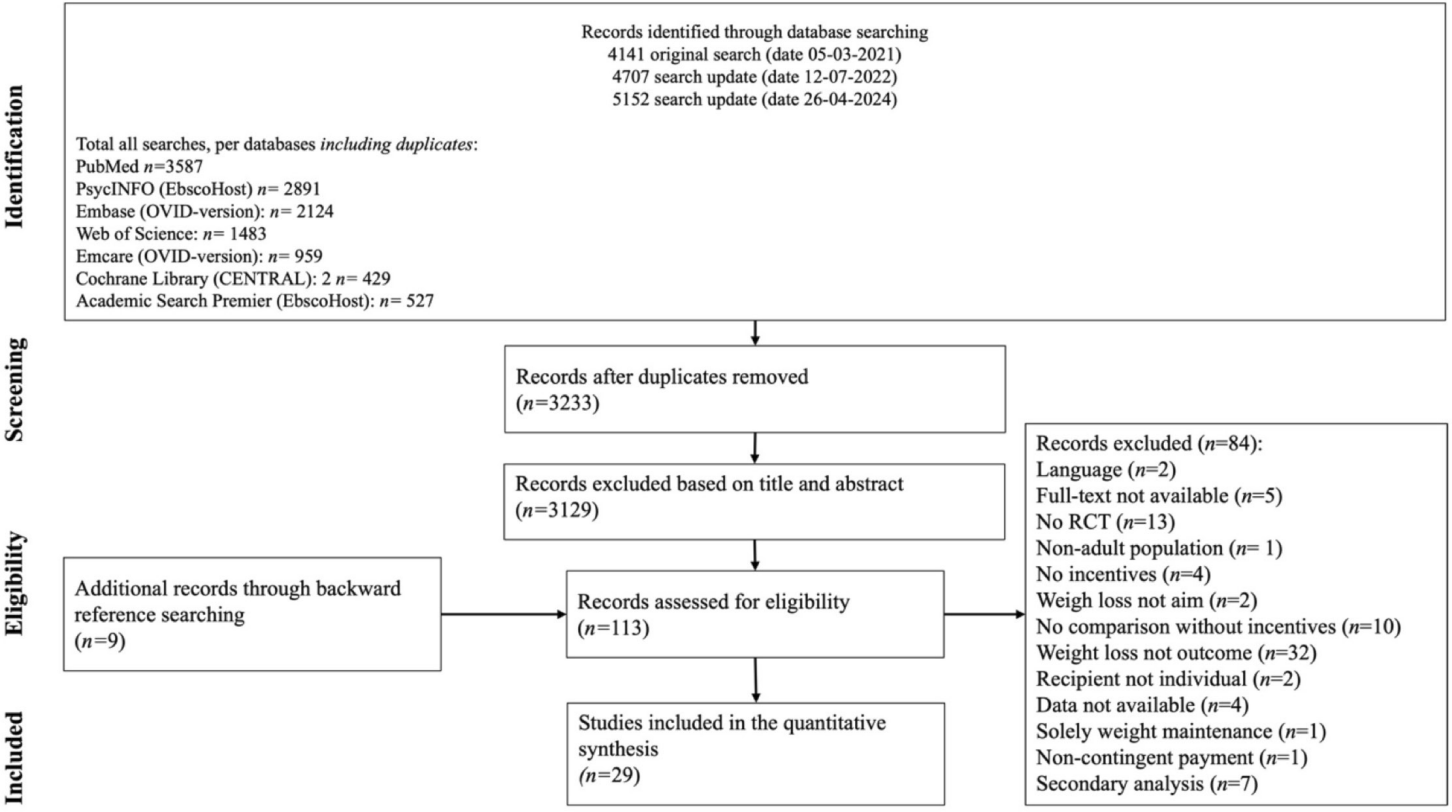
Study selection PRISMA flow chart (Page et al. 2021).

**FIGURE 2:**
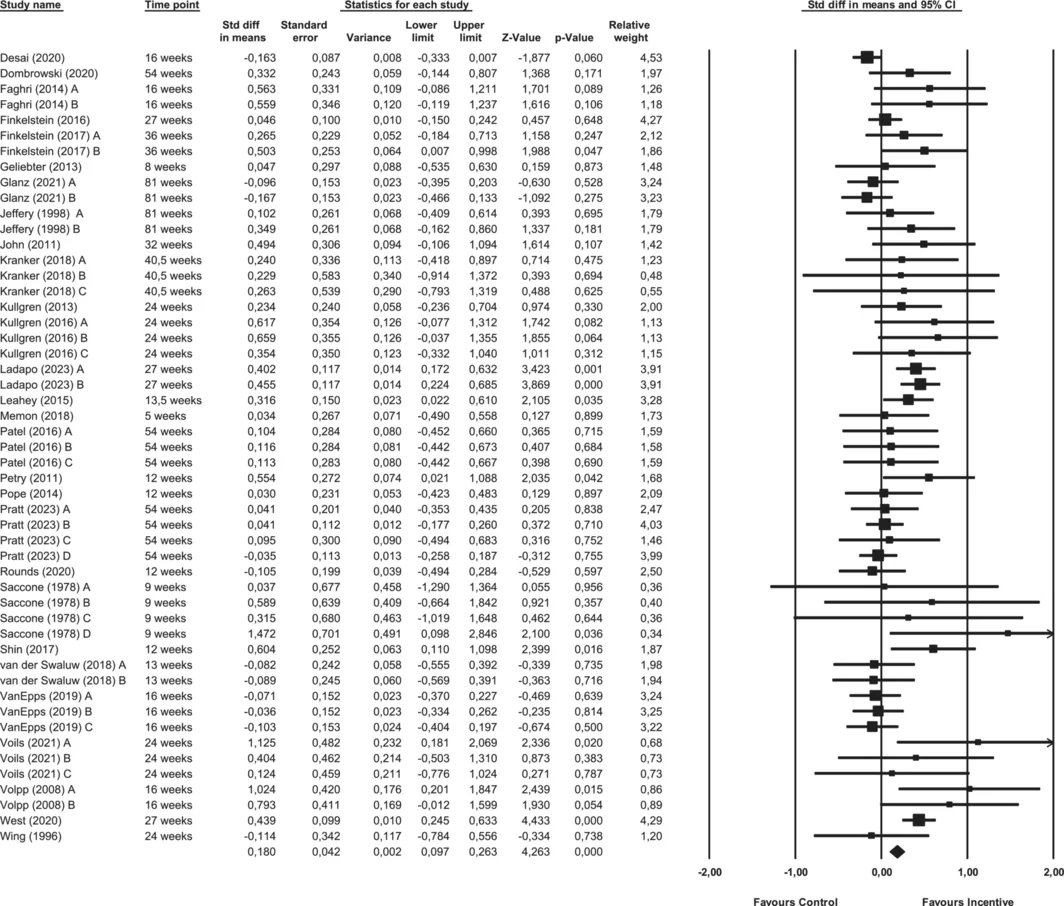
Study estimates of the effect of incentives on weight loss during the incentive phase (without Sykes‐Muskett et al. 2017).

**FIGURE 3:**
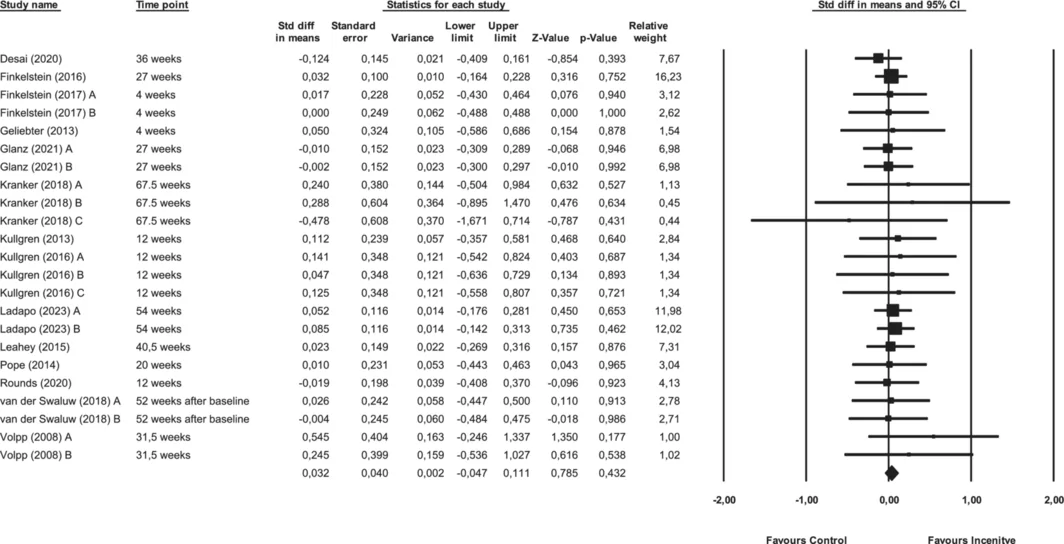
Study estimates of the effect of incentives on weight loss during the post‐incentive phase.

We identified 3233 possible articles after duplicate removal. After title and abstract screening, 113 articles (including nine found through backward reference searching) underwent full‐text screening. Eighty‐four of these were excluded. Finally, 29 articles were included in the meta‐analyses (see Figure 1), reporting 75 comparisons on the effects of incentives on weight loss during both the incentive and post‐incentive phases. These studies included a total of 2396 adults in the control condition and 3397 in the incentive condition. The characteristics of the included studies are summarized in a study characteristics table (see Supporting Information S1: Appendix A, and Figures 2 and 3).

### 3.1 Study Characteristics

A total of 29 studies were included in this meta‐analysis. Appendix A summarizes their key characteristics per condition (e.g., incentive features, control groups, incentive targets, BCTs, incentive and post‐incentive durations, outcomes, and results). Across studies, the average sample size was 169 participants (SD = 181), including individuals with obesity, overweight, diabetes, pre‐diabetes, or without chronic conditions. Incentive targets included 21 physiological outcomes (e.g., weight loss), five lifestyle behaviors (e.g., steps), 13 process behaviors (e.g., attendance), and 10 multiple targets. Of the multiple‐target conditions, four combined process behaviors with physiological outcomes, four combined lifestyle behaviors with physiological outcomes, and two combined lifestyle with process behaviors.

In most conditions (39), participants were guaranteed to receive the incentive if they met the target, whereas five offered a certain chance and four an uncertain chance. Cash incentives were most common (22 conditions), followed by lotteries (10) and deposit contracts (10). The average incentive magnitude was $737.1 (SD = 1015.1). The average incentive phase duration was 31.7 weeks (SD = 23.8), and the average post‐incentive duration was 27.6 weeks (SD = 10.1).

### 3.2 Overall Analysis: Incentive Effect and Heterogeneity Assessment

#### 3.2.1 Incentive Phase

Fifty‐two comparisons from 29 studies showed a very‐small‐to‐small, positive, and significant effect of financial incentives on weight loss during the incentive phase (SMD = 0.168, *p* < 0.001, 95% CI [0.073–0.263]; see Supporting Information S1: Appendix B, Figure B1); also after the removal of one outlier [59] (SMD = 0.180, *p* < 0.001, 95% CI [0.097–0.263]; see Figure 2), with a moderate level of heterogeneity (*I* ^2^ = 44.28%; *Q* (50) = 89.74, *p* < 0.001) [60].

#### 3.2.2 Post‐Incentive Phase

Of the 29 studies, 14 studies (23 comparisons) reported follow‐up measurements post‐incentive. No significant incentive effect on weight loss was found (SMD = 0.032, *p* = 0.432, 95% CI [−0.047–0.111]; see Figure 3) with no heterogeneity (*Q* (22) = 5.02, *p* = 1.000; *I* ^2^ = 0.00%) [60].

### 3.3 Subgroup Analysis: Incentive Target

#### 3.3.1 Incentive Phase

In line with our hypothesis, the effect of incentives on weight loss in the incentive phase differed significantly by incentive target (*Q* (3) = 8.78, *p* = 0.032, *I* ^2^ = 44.28%). The within‐group analysis showed that interventions incentivizing multi‐target and physiological outcomes (i.e., weight loss) both showed a significant effect, compared to the nonsignificant effects of lifestyle (e.g., physical activity) and process behavior (e.g., gym attendance), with multi‐target interventions demonstrating the largest effect size (see Table 1, Supporting Information S1: Appendix B, Figure B2). However, as the lifestyle behavior group contains a small number of comparisons, this may have reduced its statistical power.

**TABLE 1:** Moderator analysis incentive target for the incentive phase.

Incentive target	SMD	*p*	95% CI	Number of comparisons
Physiological outcome	0.228	0.001	[0.098 to 0.357]	23
Lifestyle behavior	0.134	0.342	[−0.143 to 0.412]	5
Process behavior	0.020	0.712	[−0.086 to 0.126]	13
Multi‐target	0.317	0.008	[0.082 to 0.551]	10

#### 3.3.2 Post‐Incentive Phase

Contrary to our hypothesis, the effect of incentives on weight loss in the post‐incentive phase did not differ significantly by incentive target (*Q* (3) = 0.65, *p* = 0.884, *I* ^2^ = 0.00%), possibly due to the small number of comparisons (i.e., 23) reducing statistical power (see Table 2, Supporting Information S1: Appendix B, Figure B3). This aligns with the absence of overall heterogeneity in the post‐incentive phase (*I* ^2^ = 0.00%), leaving no variance to explain.

**TABLE 2:** Moderator analysis incentive target for the post‐incentive phase.

Incentive target	SMD	*p*	95% CI	Number of comparisons
Physiological outcomes	0.060	0.307	[−0.055 to 0.174]	12
Lifestyle behavior	0.038	0.697	[−0.155 to 0.232]	2
Process behavior	0.017	0.894	[−0.232 to 0.266]	4
Multi‐target	−0.019	0.814	[−0.174 to 0.137]	5

### 3.4 Subgroup Analysis: Type of BCT

#### 3.4.1 Incentive Phase

Contrary to our hypothesis, the effect of incentives on weight loss in the incentive phase did not differ significantly by type of BCTs (*Q* (1) = 1.84, *p* = 0.178, *I* ^2^ = 44.28%). However, the within‐group analysis showed a small, significant positive effect of interactive BCTs (i.e., requiring active participation from participants) compared to the non‐significant effect of passive BCTs (see Table 3, Supporting Information S1: Appendix B, Figure B4).

**TABLE 3:** Moderator analysis type of BCT for the incentive phase.

Type of BCT	SMD	*p*	95% CI	Number of comparisons
Interactive	0.213	0.000	[0.103 to 0.322]	36
Passive	0.104	0.072	[−0.009 to 0.218]	15

#### 3.4.2 Post‐Incentive Phase

Contrary to our hypothesis, the effect of incentives on weight loss in the post‐incentive phase did not differ significantly by type of BCT (*Q* (1) = 0.04, *p* = 0.846, *I* ^2^ = 0.00%) (see Table 4, Supporting Information S1: Appendix B, Figure B5). This may be due to the small number of studies and aligns with the absence of overall heterogeneity in the post‐incentive phase.

**TABLE 4:** Moderator analysis type of BCT for the post‐incentive phase.

Type of BCT	SMD	*p*	95% CI	Number of comparisons
Interactive	0.026	0.596	[−0.070 to 0.123]	14
Passive	0.043	0.541	[−0.094 to 0.180]	9

### 3.5 Risk of Bias

Bias was assessed according to the Risk of Bias Cochrane tool (see Supporting Information S1: Appendix C) [55]. Many studies did not provide enough details to adequately assess random sequence generation (*n* = 13), allocation concealment (*n* = 13), blinding outcome assignments (*n* = 12), incomplete outcome data (*n* = 4), selective reporting (*n* = 13), or other bias (*n* = 7) leading to an unclear risk of bias. Random sequence generation (*n* = 2), allocation concealment (*n* = 1), blinding outcome assignments (*n* = 6), incomplete outcome data (*n* = 5), selective reporting (*n* = 2), and other bias (*n* = 4) were potential sources of bias in some studies (i.e., high risk of bias). However, because of the nature of health behavior change interventions with financial incentives it is not possible to blind participants; therefore, this risk of bias was not evaluated [19].

### 3.6 Publication Bias

For the incentive phase, the funnel plot (see Supporting Information S1: Appendix D, Figure D1) showed left‐side asymmetry, and the trim‐and‐fill procedure estimated 13 missing studies. The rank correlation test showed significant left‐sided funnel plot asymmetry, indicating some publication bias (Tau = 0.296, *p* = 0.001). However, the fail‐safe N indicated that 455 studies averaging a z‐value of zero should be added to the analysis to make the overall effect size statistically insignificant [58]. For the post‐incentive phase, the funnel plot (see Supporting Information S1: Appendix D, Figure D2) showed left‐side asymmetry, and the trim‐and‐fill procedure estimated four missing studies. Significant funnel plot asymmetry was found (Tau = 0.304, *p* = 0.021), and the fail‐safe N was 23 studies. Since there is some evidence of publication bias for both the incentive and post‐incentive phases, the results should be interpreted with caution [58].

## 4 Discussion

This meta‐analysis assessed the effect of financial incentives in weight loss interventions during the incentive (when participants received incentives) and post‐incentive (after incentive removal) phases. Financial incentives were effective in promoting weight loss during the incentive phase, albeit with a very‐small‐to‐small effect, but not in the post‐incentive phase. The overall effect size of the incentives on the short‐term fall within the very‐small‐to‐small range according to Cohen's d threshold [61]. Therefore, the clinical relevance could possibly be limited. In the incentive phase, participants lost more weight in interventions that incentivized physiological outcomes (i.e., weight loss) or multiple targets, compared to those that incentivized lifestyle or process behaviors. In the post‐incentive phase, no differences were observed between interventions with different incentive targets. Overall, no differences were found between interventions combining incentives with passive versus active BCTs; however, within‐subgroup analysis indicated that interventions combining incentives only with passive BCTs appeared ineffective for weight loss. However, the non‐significant moderator results should be interpreted cautiously, as it is unclear whether they reflect a true absence of effect or insufficient power to detect small differences. Financial incentive interventions may be effective for short‐term weight loss, yet evidence for long‐term effects remains limited, indicating that possibly incentives alone are unlikely to ensure sustainable weight loss.

The findings suggest that incentives promote short‐term weight loss (incentive phase) but not after their removal. This aligns with previous research on incentives for health behaviors, which found them effective during incentive provision but not after their removal [12, 14, 21, 22, 23]. However, other reviews did find possible lasting effects [13, 17, 18, 29, 30]. While this meta‐analysis found no lasting post‐incentive weight loss effects, only about half of the studies reported post‐incentive results, with durations ranging from four to 67.5 weeks (*M* = 31.6, SD = 21.5). This limits conclusions about long‐term effects, highlighting the need for more studies assessing post‐incentive weight changes. Furthermore, these mixed findings may be due to differences in target populations and incentive characteristics, such as magnitude (amount of money), frequency (e.g., daily and monthly), and form (e.g., cash and voucher) [6, 24], complicating comparisons across studies [24, 25]. To illustrate, this meta‐analysis revealed substantial variation in incentive characteristics within its sample, as reflected by the moderate heterogeneity in the incentive phase. Notably, this heterogeneity largely persisted even after the subgroup analyses on incentive targets and BCTs, suggesting that additional factors contribute to differences in effectiveness. For instance, incentives varied in form (i.e., lottery, cash, voucher, and deposit) and magnitude, ranging from $14.25 [62] to $5550 [63]. The effect of these variations should be further explored in future research.

When examining incentive targets, studies incentivizing multiple targets or physiological outcomes showed a larger effect on weight loss during the incentive phase than those targeting process or lifestyle behaviors. However, the relatively small number of comparisons within subgroups may limit the findings of this subgroup analysis. Our findings indicate that targeting process behaviors is least effective for weight loss, likely because these behaviors (e.g., gym attendance) are not directly linked to weight loss. Based on the review by Paul‐Ebhohimhen and Avenell (2008) [21], we hypothesized that incentivizing behavior change (i.e., lifestyle behaviors) would be more effective than incentivizing weight loss itself (i.e., physiological outcomes); however, our findings suggest the opposite. As Burns et al. (2012) [14] suggested, the principle “you get what you pay for” may explain this finding—specifically, that incentivizing weight loss will result in weight loss, whereas incentivizing other health behaviors may not necessarily result in weight loss. However, while incentive interventions targeting lifestyle behaviors alone were not more effective, those incentivizing multiple targets, including lifestyle behaviors (*n* = 6), generally showed effectiveness, with effect sizes ranging from 0.263 to 0.604 (SMD). Incentivizing multiple targets had the largest effect in the incentive phase, supporting our hypothesis. This could be explained by the fact that achieving weight loss is a slow and complex process requiring multiple health behaviors over time (e.g., physical activity and eating healthier) [19, 27, 64]. Furthermore, a meta‐analysis on lifestyle recommendations found that a moderate number of recommendations is most effective, as it engages participants without overcomplicating the intervention [65]. Also, there is great individual variability in what works best [66], which could explain the effectiveness of incentivizing multiple targets. The included studies with multiple targets varied in their combinations, such as incentivizing dietary self‐monitoring through food logging (process) and weight loss (physiological) [67], or incentivizing increasing physical activity (lifestyle) and weight loss (physiological) [68]. However, the most effective combination of incentive targets remains unclear. Future research should explore the most (cost‐)effective incentive target combinations, possibly through a network meta‐analysis [69].

Combining financial incentives with interactive BCTs (requiring active participation) did not result in significantly more weight loss than combining incentives with passive BCTs. In the incentive phase, however, the within‐group analysis suggested a small but significant effect for incentives combined with interactive BCTs, while the combination with passive BCTs was non‐significant. However, substantial heterogeneity remained among the studies using interactive BCTs (I^2^ = 57%), indicating considerable variation in effect sizes and potential confounding factors not accounted for in this subgroup analysis. The smaller sample size in the passive BCTs subgroup could further limit the ability to detect differences [70]. To better understand this heterogeneity, future research should explore the impact of individual BCTs on weight loss. Importantly, these findings suggest that providing passive BCTs (i.e., information provision) in combination with incentives may not be enough to affect a complex process, such as achieving weight loss. This adds to the literature showing that deposit contracts were most effective for weight loss when paired with interactive BCTs, including self‐monitoring of behavior, goal setting, feedback on performance, intention formation, and review of behavior goals [23, 42]. Furthermore, previous reviews suggest that incentives should be used within an intervention rather than as an intervention in itself [21]. Since achieving weight loss is complex [19, 27], it may be important not only to increase motivation and create opportunity by providing incentives [8, 9] but also to provide the required knowledge and skills for successful weight loss through interactive BCTs [21, 23, 36, 71]. Combining incentives with interactive BCTs may strengthen the intervention's effect by offering more tailored information and skills training, for instance, through individual coaching or personalized feedback on behavior (for review [21]). Additional meta‐regressions, including exploratory methods such as Meta‐CART [72], could help identify further sources of heterogeneity, such as incentive form or magnitude, intervention duration, or specific BCTs. These analyses were not conducted because they fall beyond the aims and scope of this review. However, such approaches represent a valuable direction for future research to clarify moderators of incentive effectiveness and to build on the present findings. Furthermore, the current shift towards eHealth and technology‐based interventions could provide accessible platforms for both incentives and interactive BCTs, with AI potentially facilitating personalization to better tailor information and skills training to individual needs.

Financial incentives can enhance opportunity by decreasing barriers [36, 37] and boost motivation by providing immediate rewards [28] to engage in health behaviors, as outlined by the COM‐B model [36]. While financial incentives impact the individual level, they may not address broader systemic factors that contribute to unhealthy behavior (e.g., limited access to healthy food). The COM‐B model also encompasses opportunity at the systemic, societal level, including factors outside of individual control, such as legislation, social norms, access to sports facilities, and neighborhood safety [37]. A pitfall of individual incentive interventions is that they often fail to account for these systemic influences on health. Future research should investigate the role of these systemic factors and how incentives can interact with them to enhance long‐term health outcomes.

### 4.1 Limitations and Future Directions

This meta‐analysis and the incentive literature, in general, have several limitations. First, publication bias was detected, warranting caution in interpreting the results. However, the classic fail‐safe N suggests that 454 (incentive phase) and 23 (post‐incentive phase) null‐effect studies would be required to reduce the effect to non‐significance [58].

Second, many studies had a high or unclear risk of bias. While we did not evaluate the blinding of participants due to the nature of incentive studies in which participant blinding is not possible [19], efforts could be made to blind experimenters and data analysts. Additionally, most studies fail to report correlations between outcomes across time points or lack standardized methods for reporting weight loss. This complicates effect size calculations and hampers comparison and aggregation of results. Therefore, this meta‐analysis conservatively assumed a zero correlation between time points, possibly underestimating the true effect. Although our risk‐of‐bias assessment suggests that methodology and reporting have improved over the years, transparency and reporting still need improvement. Future studies should adhere to risk‐of‐bias tools, such as the Cochrane Collaboration tool, and adopt standardized reporting methods. Since the inception of this study, the Cochrane risk of bias tool [57] has been updated to Revised Cochrane Risk‐of‐Bias Tool for Randomized Trials (RoB 2), which may offer more accurate assessments in future studies. In addition, we did not evaluate the overall certainty of evidence using the GRADE tool. Future research could utilize this tool to evaluate the robustness of the findings and thereby provide additional recommendations.

Third, the scarcity of post‐incentive assessments limited statistical power, particularly for the subgroup analyses. Consequently, definitive conclusions about the long‐term effectiveness of incentives for weight loss cannot be drawn. Given the persistent challenge of maintaining health behavior change [12, 25], future research should include multiple follow‐up assessments to evaluate long‐term effects.

Finally, although interventions using multiple incentive targets appeared most effective in the incentive phase, the specific incentive targets and their combinations varied across studies, hindering clear recommendations on optimal target combinations. Therefore, future research should further explore this. Personalizing these combinations may enhance (cost‐)effectiveness, as weight loss strategies often vary between individuals [66]. Furthermore, the interaction between incentive features and individual or context factors, such as socioeconomic position, remains unclear [14, 24, 25, 73]. Future research should systematically explore these relationships, potentially using approaches such as the multiphase optimization strategy (MOST) [74] and utilizing a systematic framework to describe incentive interventions [24]. MOST is a framework for systematically testing and optimizing different intervention components (such a duration, magnitude, frequency, combinations of incentives and other BCTS) to influence outcomes such as weight loss.

## 5 Conclusions

In summary, financial incentives have a small effect on weight loss while incentives are provided. However, after incentive removal, no effect on weight loss was found, although the number of studies reporting post‐incentive data was limited. Subgroup analyses suggest that incentivizing multiple targets and physiological outcomes (e.g., weight loss), is most effective while incentives are provided. Additionally, interventions combining financial incentives with non‐tailored, passive BCTs (e.g., general information provision) may be insufficient for achieving outcomes that are complex to attain like weight loss. More evidence is needed to investigate whether weight loss is continued or maintained after incentive removal. Future research should explore the most cost‐effective combinations of incentive targets and identify which interactive BCTs should be combined with incentives.

## Funding

This research was funded by ZonMw (The Netherlands Organization for Health Research and Development) and the Netherlands Cardiovascular Research Initiative: An initiative with support of the Dutch Heart Foundation, CVON2016‐12 BENEFIT, the Enduring Rewards project funded by the Netherlands Organization of Scientific Research (NWO) (project number: 403.19.246), and The Stevin Prize from NWO.

## Conflicts of Interest

The authors declare no conflicts of interest.

## Supporting information


**Table S1:** Characteristics of the included studies.

**Figure B1** Overall analysis for the incentive phase including all studies.

**Figure B2** Moderator analysis for incentive target for the incentive phase.

**Figure B3** Moderator analysis for incentive target for the post‐incentive phase.

**Figure B4** Moderator analysis for passive or interactive BCTs for the incentive phase.

**Figure B5** Moderator analysis for passive or interactive BCTs for the post‐incentive phase.

**Figure D1** Funnel plot for the incentive phase.

**Figure D2** Funnel plot for the post‐incentive phase.

**Appendix S1:** Supporting information.

## Data Availability

The data that support the findings of this study are available from the corresponding author upon reasonable request.
