# Supplementary material for: Enhancing Weight Loss Programs: A Meta‐Analysis of Financial Incentives and Behavior Change Techniques
Source: Obes Rev. 2026 Mar 18;27(10):e70124. doi: 10.1111/obr.70124 (PMC13551397; doi:10.1111/obr.70124)
Supplement: Supplementary file 2 [file OBR-27-e70124-s009.pdf]

Enhancing Weight Loss Programs: A Meta-Analysis of Financial Incentives and Behavior Change Techniques

Sara M. Hondmann[a*], Milon H. M. van Vliet[b, c*], Roxy A. van Eersel[a], Linda D. Breeman[a], Mike Keesman[a], David R. de Buisonjé[a, h, j], George Haroon[a], Roderik Kraaijenhagen[e], Veronica R. Janssen[d], Andrea W. M. Evers[a, f, g †]

* Authors contributed equally

[a] Health, Medical and Neuropsychology Unit, Institute of Psychology, Leiden University, Leiden, The Netherlands

[b] Department of Public Health and Primary Care, Leiden University Medical Center, Leiden, the Netherlands

[c] National eHealth Living Lab, Leiden University Medical Center, Leiden, the Netherlands

[d] Department of Cardiology, Leiden University Medical Center, Leiden, The Netherlands

[e] Hearts4People Foundation, Amsterdam, The Netherlands

[f] Department of Psychiatry, Leiden University Medical Center, Leiden, The Netherlands

[g] Medical Delta, Leiden University, Delft University of Technology, Erasmus University, Delft, The Netherlands

[h] Department of Human-Centered Design, Faculty of Industrial Design Engineering, Delft University of Technology, Delft, The Netherlands.

[i] Department of Economics and Business Economics, Radboud school of Management, Radboud University, Nijmegen, The Netherlands.

†04-08-2025

Corresponding Author: Sara M. Hondmann,

Health, Medical and Neuropsychology Unit, Institute of Psychology, Leiden University Wassenaarseweg 52 2333 AK Leiden, The Netherlands

E-mail s.m.hondmann@fsw.leidenuniv.nl

Appendix A

Characteristics of the included studies

First Author (year)	Total N *n per condition* Study sample	Incentive characteristics	Control Group	Incentive target	Type of BCT	Duration incentive phase	Duration post-incentive phase	Outcome & measurement	Results *Incentive phase (Baseline – T1)* *Post-incentive phase (T1-T2)*
Desai (2020)	N = 213<br>*Control:* 71<br>*Incentive:* 142<br>obese adults	*Form:* Voucher/coupon for free service(s) or good(s)<br>*Magnitude:* $520<br>*Certainty:* Certain<br>*Participation incentive*<br>- *incentive phase*: No<br>- *post-incentive phase:* Yes	No incentives: Diabetes prevention program	Multiple - Process behavior & physiological outcome	Interactive	16 weeks	36 weeks	Relative weight loss in percentage, measured as BMI & objectively verified	*Incentive phase*<br>Control: M = -3.4%, SE = .33<br>Incentive: M = -2,6%, SE = .28<br><br>*Post-incentive phase*<br>Control: M = -7.1%, SE = 3.60<br>Incentive: M = -3.7%, SE = 1.05
Dombrowski (2020)	N = 69<br>*Control:* 33<br>*Incentive:* 36<br>obese adults	*Form:* Cash<br>*Magnitude:* $492<br>*Certainty:* Certain<br>*Participation incentive*<br>- *incentive phase*: Yes	No incentives: Narrative text message only	Physiological outcome	Passive	54 weeks	-	Absolute weight loss in kilograms & objectively verified	*Incentive phase*<br>Control: M = -1.33, SE = .88<br>Incentive: M = -2.98, SE = .82
Faghri (2014) **AB**	N = 73<br>*Control:* 38	**A**: *Form:* Cash<br>*Magnitude*: $160<br>*Certainty:* Certain<br>*Participation incentive*	No incentives:	Physiological outcome	Interactive	16 weeks	-	Absolute weight loss in pounds & unknown	**A:** *Incentive phase*<br>Control: M = 0, SE = 0<br>Incentive: M = -5.92, SE = 3.41

	Incentive: 35<br>(**A:** 19; **B:** 16)<br>obese adults	- *incentive phase*: Yes<br><br>**B:** *Form:* Deposit contract<br>*Magnitude:* $320<br>*Certainty:* Certain<br>*Participation incentive*<br>- *incentive phase:* Unknown	Worksite weight loss program						**B**: *Incentive phase*<br>Control: M = 0, SE = 0<br>Incentive: M = -4.45, SE = 2.95
Finkelstein (2016)	N = 400<br>*Control:* 203<br>*Incentive:* 197<br>healthy adults	*Form:* Cash<br>*Magnitude*: $588.60<br>*Certainty:* Certain<br>*Participation incentive*<br>- *incentive phase:* Yes<br>- *post-incentive phase:* Yes	No incentives: Activity tracker & educational materials	Lifestyle behavior	Passive	27 weeks	27 weeks	Absolute weight loss in kilograms & objectively verified	*Incentive phase*<br>Control: M = +.20, SE = .38<br>Incentive: M = +.40, SE = .36<br><br>*Post-incentive phase*<br>Control: M = -.40, SE = .84<br>Incentive: M = -.60, SE = .79
Finkelstein (2017) **AB**	N = 161<br>*Control:* 54<br>*Incentive:* 107<br>(**A:** 67; **B:** 40)<br>overweight adults	**A**: *Form:* Cash<br>*Magnitude:* $475.20<br>*Certainty:* Certain<br>*Participation incentive*<br>- *incentive phase*: Yes<br>- *post-incentive phase*: Yes<br><br>**B**: *Form:* Lottery<br>*Magnitude:* $4725<br>*Certainty:* Certain chance<br>*Participation incentive*<br>- *incentive phase:* Unknown<br>- *post-incentive phase:* No	No incentives: Activity tacker & lifestyle program	Multiple - Lifestyle behavior & physiological outcome	Interactive	36 weeks	4 weeks	Absolute weight loss in kilogram & objectively verified	**A:** *Incentive phase*<br>Control: M = -1.8, SE = .51<br>Incentive: M = -3.0, SE = .59<br><br>*Post-incentive phase*<br>Control: M = -.80, SE = .56<br>Incentive: M = -2.1, SE = .64<br><br>**B:** *Incentive phase*<br>Control: M = -1.8, SE = .51<br>Incentive: M = -3.4, SE = .43

									Post-incentive phase<br>Control: M = -.80, SE = .56<br>Incentive: M = -2.4, SE = .46
Geliebter (2013)	N = 47<br>*Control:* 19<br>*Incentive:* 28<br>obese adults	*Form:* Voucher/coupon for discount<br>*Magnitude:* 50% discount<br>*Certainty:* Certain<br>*Participation incentive*<br>*- incentive phase:* Unknown<br>*- post-incentive phase:* Unknown	No incentives: Educational materials	Process behavior	Passive	8 weeks	4 weeks	Absolute weight in kilogram & objectively verified	*Baseline*<br>Control: M = 84.6, SE = 3.78<br>Incentive: M = 82.7, SE = 3.24<br><br>*(Final timepoint of) Incentive phase*<br>Control: M = 85.0, SE = 4.33<br>Incentive: M = 81.9, SE = 3.47<br><br>*(Final timepoint of) Post-incentive phase*<br>Control: M = 84.4, SE = 4.13<br>Incentive: M = 80.0, SE = 3.44
Glanz (2021) **A**	N = 172<br>*Control:* 86<br>*Incentive:* 86<br>obese adults	*Form:* Lottery<br>*Magnitude:* $1701<br>*Certainty:* Certain chance<br>*Participation incentive*<br>*- incentive phase:* Yes<br>*- post-incentive phase*: Yes	No incentives: Workplace wellness program	Physiological outcome	Interactive	81 weeks	27 weeks	Absolute weight in pounds & objectively verified	*Baseline**<br>Control: M = 228.7, SE = 4.4<br>Incentive: M = 236.9, SE = 3.6<br>*(Final timepoint of) Incentive phase*<br>Control: M = 227.3, SE = 4.8

									Incentive: M = 230.2, SE = 3.9<br><br>*(Final timepoint of) Post-incentive phase*<br>Control: M = 229.4, SE = 4.9<br>Incentive: M = 232.9, SE = 4.0
Glanz (2021) **B**	N = 172<br>*Control:* 86<br>*Incentive:* 86 obese adults	*Form:* Lottery<br>*Magnitude:* $1701<br>*Certainty:* Certain chance<br>*Participation incentive*<br>*- incentive phase:* Yes<br>*- post-incentive phase:* Yes	No incentives: Environmental strategies	Physiological outcome	Interactive	81 weeks	27 weeks	Absolute weight in pounds & objectively verified	*Baseline**<br>Control: M = 233.6, SE = 4.7<br>Incentive: M = 236.7, SE = 4.5<br><br>*(Final timepoint of) Incentive phase*<br>Control: M = 230.1, SE = 4.7<br>Incentive: M = 232.8, SE = 5.0<br><br>*(Final timepoint of) Post-incentive phase*<br>Control: M = 229.7, SE = 4.0<br>Incentive: M = 232.5, SE = 4.9
Jeffery (1998) **A**	N = 59<br>*Control*: 31<br>*Incentive*: 28 obese adults	*Form:* Cash<br>*Magnitude:* $491<br>*Certainty:* Certain<br>*Participation incentive*	No incentive: Standard behavior	Process behavior	Interactive	81 weeks	-	Absolute weight loss in kilograms &	*Incentive phase*<br>Control: M = -3.8, SE = 1.30

		- *incentive phase:* Unknown	therapy & supervised walks					objectively verified	Incentive: M = -4.5, SE = 1.20
Jeffery (1998) **B**	N = 60<br>*Control:* 32<br>*Incentive*: 28<br>obese adults	*Form:* Cash<br>*Magnitude:* $491<br>*Certainty:* Certain<br>*Participation incentive*<br>*- incentive phase:* Unknown	No incentive: Standard behavior therapy, supervised walks & personal trainer	Process behavior	Interactive	81 weeks	-	Absolute weight loss in kilograms & objectively verified	*Incentive phase*<br>Control: M = -2.9, SE = 1.10<br>Incentive: M = -5.1, SE = 1.30
John (2011)	N = 44<br>*Control:* 22<br>*Incentive:* 22<br>obese adults	*Form:* Deposit contract<br>*Magnitude:* $653.80<br>*Certainty:* Certain<br>*Participation incentive*<br>*- incentive phase:* Yes	No incentives: Weight-monitoring program	Physiological outcome	Interactive	32 weeks	-	Absolute weight loss in pounds & self-reported	*Incentive phase*<br>Control: M = -1.17, SE = 2.94<br>Incentive: M = -7.75, SE = 2.73
Kranker (2018) **ABC**	N = 66<br>*Control:* 27<br>*Incentive:* 39<br>(**A:** 31; **B:** 4; **C:** 4)<br>adults with diabetes	**A:** *Form:* Voucher/coupon for free services(s) or good(s)<br>*Magnitude:* $200<br>*Certainty:* Certain<br>*Participation incentive*<br>*- incentive phase:* Unknown<br>*- post-incentive phase:* Unknown<br><br>**B:** *Form:* Voucher/coupon for free services(s) or good(s)	No incentives: Education materials & self-management support	**A:** Physiological outcome<br><br>**B:** Lifestyle behavior<br><br>**C:** Multiple - Lifestyle behavior & physiological outcome	Interactive	40.5 weeks	67.5 weeks	Absolute weight loss in pounds & objectively verified	**A:** *Incentive phase*<br>Control: M = 0, SE = 0<br>Incentive: M = -2.13, SE = 1.53<br><br>*Post-incentive phase*<br>Control: M = 0, SE = 0<br>Incentive: M = -5.45, SE = 2.15<br><br>**B:** *Incentive phase*<br>Control: M = 0, SE = 0<br>Incentive: M = -.10, SE = 4.59

		Magnitude: $200<br>*Certainty:* Certain<br>*Participation incentive*<br>*- incentive phase:* Unknown<br>*- post-incentive phase:* Unknown<br><br>**C**: *Form:* Voucher/coupon for free services(s) or good(s)<br>*Magnitude:* Unknown<br>*Certainty:* Certain<br>*Participation incentive*<br>*- incentive phase:* Unknown<br>*- post-incentive phase:* Unknown							*Post-incentive phase*<br>Control: M = 0, SE = 0<br>Incentive: M = -3.02, SE = 4.94<br><br>**C:** *Incentive phase*<br>Control: M = 0, SE = 0<br>Incentive: M = -2.97, SE = 9.56<br><br>*Post-incentive phase*<br>Control: M = 0, SE = 0<br>Incentive: M = +2.64, SE = 3.76
Kullgren (2013)	N = 70<br>*Control*: 35<br>*Incentive:* 35<br>obese adults	*Form:* Cash<br>*Magnitude:* $600<br>*Certainty:* Certain<br>*Participation incentive*<br>*- incentive phase:* Yes<br>*- post-incentive phase:* Yes	No incentive: Educational materials & monthly weigh-ins with reminders	Physiological outcome	Passive	24 weeks	12 weeks	Absolute weight loss in kilograms & objectively verified	*Incentive phase*<br>Control: M = -.50, SE = .89<br>Incentive: M = -1.70, SE = .84<br><br>*Post-incentive phase*<br>Control: M = -.40, SE = .84<br>Incentive: M = -.80 SE = .84
Kullgren (2016)<br>**ABC**	N = 132<br>*Control:* 33<br>*Incentive:* 99 (**A:** 33; **B:** 33; **C:** 33)	**A**: *Form:* Deposit<br>*Magnitude:* $504<br>*Certainty:* Certain<br>*Participation incentive*<br>*- incentive phase*: Yes	No incentives: Educational materials & monthly	Physiological outcome	Passive	24 weeks	12 weeks	Absolute weight loss in pounds & objectively verified	**A**: *Incentive phase*<br>Control: M = +1.0, SE = 1.32<br>Incentive: M = -4.3, SE = 1.5

	obese adults	- *post-incentive phase:* Yes<br><br>**B**: *Form:* 1:1 matched deposit<br>*Magnitude:* $1008<br>*Certainty:* Certain<br>*Participation incentive*<br>- *incentive phase:* Yes<br>- *post-incentive phase:* Yes<br><br>**C**: *Form:* 2:1 matched deposit<br>*Magnitude:* $1512<br>*Certainty*: Certain<br>*Participation incentive*<br>- *incentive phase:* Yes<br>- *post-incentive phase:* Yes	weigh-ins with reminders						*Post-incentive phase*<br>Control: M = +2.1, SE = 1.38<br>Incentive: M = -5.1, SE = 1.93<br><br>**B:** *Incentive phase*<br>Control: M = +1.0, SE = 1.32<br>Incentive: M = -5.3, SE = 1.76<br><br>*Post-incentive phase*<br>Control: M = +2.1, SE = 1.38<br>Incentive: M = -3.6, SE = 1.67<br><br>**C:** *Incentive phase*<br>Control: M = +1.0, SE = 1.32<br>Incentive: M = -2.3, SE = 1.7<br><br>*Post-incentive phase*<br>Control: M = +2.1, SE = 1.38<br>Incentive: M = -2.8, SE = 1.76
Ladapo (2022) **AB**	N = 668	**A:** *Form:* Cash<br>*Magnitude:* $750	No incentives:	**A:** Multiple -Lifestyle	Interactive	27 weeks	54 weeks	Absolute weight loss in	**A:** *Incentive phase*

	Control: 221<br>*Incentive:* 447 (**A:** 222; **B:** 225) obese adults	*Certainty:* Certain<br>*Participation incentive*<br>*- incentive phase:* Yes<br>*- post-incentive phase:* Yes<br><br>**B:** *Form:* Cash<br>*Magnitude:* $750<br>*Certainty:* Certain<br>*Participation incentive*<br>*- incentive phase:* Yes<br>*- post-incentive phase:* Yes	Weight-loss program membership, self-monitoring tools, educational materials & monthly check-in	behavior & process behavior<br><br>**B:** Physiological outcome				kilograms & objectively verified	Control: M = -2.21, SE = .38<br>Incentive: M = -4.47, SE = .38<br><br>*Post-incentive phase*<br>Control: M = -2.74, SE = .39<br>Incentive: M = -5.43, SE = .41<br><br>**B:** *Incentive phase*<br>Control: M = -2.21, SE = .38<br>Incentive: M = -4.79, SE = .38<br><br>*Post-incentive phase*<br>Control: M = -2.74, SE = .39<br>Incentive: M = -4.61, SE = .41
Leahey (2015)	N = 180<br>*Control:* 91<br>*Incentive:* 89 obese adults	*Form:* Lottery<br>*Magnitude:* $100<br>*Certainty:* Uncertain chance<br>*Participation incentive*<br>*- incentive phase:* Yes<br>*- post-incentive phase:* Yes	No incentive: Community wellness program & online behavioral weight loss program	Physiological outcome	Interactive	13.5 weeks	40.5 weeks	Relative weight loss in percentage, measured in kilograms & objectively verified	*Incentive phase*<br>Control: M = -4.3% SE = .66<br>Incentive: M = -6.4%, SE = .66<br><br>*Post-incentive phase*<br>Control: M = -1.2%, SE = .92

									Incentive: M = -3.1%, SE = .66
Memon (2018)	N = 56<br>*Control:* 28<br>*Incentive:* 28 overweight adults	*Form:* Cash<br>*Magnitude:* $14.25<br>*Certainty:* Certain<br>*Participation incentive - incentive phase:* Unknown	No incentives: Step counting smartphone application	Lifestyle behavior	Passive	5 weeks	-	Absolute weight in kilograms & unknown	*Baseline**<br>Control: M = 72.1, SE = 1.66<br>Incentive: M = 68.7, SE = 1.42<br><br>*(Final timepoint of) Incentive phase*<br>Control: M = 70.97 SE = 1.63<br>Incentive: M = 67.96, SE = 1.39
Patel (2016) **ABC**	N = 197<br>*Control:* 50<br>*Incentive:* 147<br>(**A:** 49; **B:** 48; **C:** 50) obese adults	**A**: *Form*: Voucher/coupon for discount, delayed<br>*Magnitude*: $550<br>*Certainty:* Certain<br>*Participation incentive - incentive phase:* Yes<br><br>**B**: *Form:* Voucher/coupon for discount, immediate<br>*Magnitude:* $550<br>*Certainty:* Certain<br>*Participation incentive - incentive phase:* Yes<br><br>**C**: *Form:* Lottery<br>*Magnitude*: $550<br>*Certainty*: Certain chance<br>*Participation incentive*	No incentives: Weight loss goal	Physiological outcome	Passive	54 weeks	-	Absolute weight loss in pounds & objectively verified	**A:** *Incentive phase*<br>Control: M = +0.1, SE = 2.07<br>Incentive: M = -1.2, SE = 1.68<br><br>**B:** *Incentive phase*<br>Control: M = +0.1, SE = 2.07<br>Incentive: M = -1.4, SE = 1.79<br><br>**C:** *Incentive phase*<br>Control: M = +0.1, SE = 2.07<br>Incentive: M = -1.0, SE = 1.07

		- *incentive phase*: Yes							
Petry (2011)	N = 56<br>*Control*: 28<br>*Incentive*: 28<br>obese adults	*Form:* Lottery<br>*Magnitude:* $504 (estimation)<br>*Certainty:* Certain chance<br>*Participation incentive*<br>*- incentive phase:* Unknown	No incentives: Educational materials & supportive counseling	Multiple – Lifestyle & process behavior	Interacti ve	12 weeks	-	Relative weight loss in percentage, measured in kilograms & objectively verified	*Incentive phase*<br>Control: M = -3.5%, SE = .77<br>Incentive: M = -6.0% SE = .93
Pope (2014)	N = 75<br>*Control*: 38<br>*Incentive:* 37<br>healthy adults	*Form:* Cash<br>*Magnitude:* $310.50<br>*Certainty:* Certain<br>*Participation incentive*<br>*- incentive phase:* Yes<br>*- post-incentive phase:* Yes	No incentives: Educational materials & fitness-center attendance goal	Process behavior	Passive	12 weeks	20 weeks	Absolute weight in kilograms & objectively verified	*Baseline**<br>Control: M = 69.1, SE = 2.3<br>Incentive: M = 69.6, SE = 2.3<br><br>*(Final timepoint of)*<br>*Incentive phase*<br>Control: M = 70.3, SE = 2.3<br>Incentive: M = 70.2, SE = 2.3<br><br>*(Final timepoint of)*<br>*Post-incentive phase*<br>Control: M = 70.7, SE = 2.3<br>Incentive: M = 70.4, SE = 2.3
Pratt (2023) **A**	N = 99<br>*Control*: 48<br>*Incentive*: 51<br>obese adults	*Form:* Cash<br>*Magnitude:* $780<br>*Certainty:* Certain<br>*Participation incentive*<br>*- incentive phase*: Yes	No incentives: Gym membership	Process behavior	Passive	54 weeks	-	Absolute weight in pounds & objectively verified	*Baseline*<br>Control: M = 219, SE = 5.04<br>Incentive: M = 219.04, SE = 4.57<br><br>*Incentive phase*

									Control: M = 223.3, SE = 6.5<br>Incentive: M = 220.7, SE = 6.6
Pratt (2023) **B**	N = 321<br>*Control:*161<br>*Incentive:* 160<br>obese adults	*Form:* Cash<br>*Magnitude:* $780<br>*Certainty:* Certain<br>*Participation incentive - incentive phase:* Yes	No incentives: In Shape program: (personal and group coaching)	Process behavior	Interactive	54 weeks	-	Absolute weight in pounds & objectively verified	*Baseline**<br>Control: M = 236, SE = 3.59<br>Incentive: M = 225.66, SE = 3.10<br><br>*(Final timepoint of) Incentive phase*<br>Control: M = 227.3, SE = 5.28<br>Incentive: M = 220.5, SE = 4.28
Pratt (2023) **C**	N = 47<br>*Control:*18<br>*Incentive:* 29<br>obese adults	*Form:* Cash<br>*Magnitude:* $520<br>*Certainty:* Certain<br>*Participation incentive - incentive phase:* Yes	No incentives: Weight Watchers: (weight Loss support group & educational materials)	Process behavior	Interactive	54 weeks	-	Absolute weight in pounds & objectively verified	*Baseline**<br>Control: M = 244, SE = 12.19<br>Incentive: M = 231.15, SE = 8.84<br><br>*(Final timepoint of) Incentive phase*<br>Control: M = 251.8, SE = 24.65<br>Incentive: M = 228.9, SE = 11.74

Pratt (2023) **D**	N = 311<br>*Control*: 155<br>*Incentive:* 156<br>obese adults	*Form:* Cash<br>*Magnitude:* $1300<br>*Certainty:* Certain<br>*Participation incentive:*<br>- *incentive phase:* Yes	No incentives: In Shape program & Weight Watchers	Process behavior	Interactive	54 weeks	-	Absolute weight in pounds & objectively verified	*Baseline**<br>Control: M = 227.6, SE = 3.52<br>Incentive: M = 233.15, SE = 3.64<br><br>*(Final timepoint of) Incentive phase*<br>Control: M = 220.4, SE = 4.62<br>Incentive: M = 228.9, SE = 5.09
Rounds (2020)	N = 102<br>*Control:* 48<br>*Incentive:* 54<br>obese adults	*Form:* Cash<br>*Magnitude:* $312<br>*Certainty:* Certain<br>*Participation incentive*<br>- *incentive phase:* Yes<br>- *post-incentive phase:* Yes	No incentives: “Gutbuster” program (weight loss program & educational materials)	Physiological outcome	Interactive	12 weeks	12 weeks	Absolute weight in pounds & objectively verified	*Baseline**<br>Control: M = 217.4, SE = 5.61<br>Incentive: M = 223.8, SE = 5.66<br><br>*(Final timepoint of) Incentive phase*<br>Control: M = 213.6, SE = 5.94<br>Incentive: M = 214.0, SE = 5.43<br><br>*(Final timepoint of) Post-incentive phase*<br>Control: M = 213.9, SE = 6.05<br>Incentive: M = 215.4, SE = 5.51

Saccone (1978) **AC**	N = 22<br>*Control*: 6<br>*Incentive*: 16 (**A:** 8; **C:** 8) overweight adults	**A**: *Form:* Deposit contract<br>*Magnitude:* $35<br>*Certainty:* Certain<br>*Participation incentive:*<br>*- incentive phase*: No<br><br>**C**: *Form:* Deposit contract<br>*Magnitude:* $35<br>*Certainty:* Certain<br>*Participation incentive*<br>*- incentive phase:* No	No incentive: Educational materials & monitoring weight	Physiological outcome	Interactive	9 weeks	-	Absolute weight loss in pounds & unknown	**A:** *Incentive phase*<br>Control: M = -5.1, SE = 1.84<br>Incentive: M = -5.3, SE = 2.09<br><br>**C:** *Incentive phase*<br>Control: M = -5.1, SE = 1.18<br>Incentive: M = -6.9, SE = 2.23
Saccone (1978) **BD**	N = 22<br>*Control:* 8<br>*Incentive:* 14 (**B:** 7; **D:** 7) overweight adults	**B**: *Form:* Deposit contract<br>*Magnitude:* $35<br>*Certainty:* Certain<br>*Participation incentive*<br>*- incentive phase:* No<br><br>**D**: *Form:* Deposit contract<br>*Magnitude:* $35<br>*Certainty:* Certain<br>*Participation incentive:*<br>*- incentive phase:* No	No incentive: Educational materials & monitoring eating behavior	Lifestyle behaviors	Interactive	9 weeks	-	Absolute weight loss in pounds & unknown	**B:** *Incentive phase*<br>Control: M = -3.7, SE = 2.62<br>Incentive: M = -7.6, SE = 2.34<br><br>**D:** *Incentive phase*<br>Control: M = -3.7, SE = 2.62<br>Incentive: M = -13.0, SE = 2.15
Shin (2017)	N = 66<br>*Control:* 34<br>*Incentive:* 32 overweight adults	*Form:* Cash<br>*Magnitude:* $278.3<br>*Certainty:* Certain<br>*Participation incentive*<br>*- incentive phase:* Yes	No incentives: Accelerometer & smartphone application	Multiple - Lifestyle behaviors & physiological outcome	Interactive	12 weeks	-	Absolute weight loss in kilograms & objectively verified	*Incentive phase*<br>Control: M = -1.1, SE = .50<br>Incentive: M = -3.1, SE = .65

Sykes-Muskett (2017)	N = 41<br>*Control*: 19<br>*Incentive:* 22<br>obese adults	*Form:* Deposit contract<br>*Magnitude:* Missing<br>*Certainty:* Certain<br>*Participation incentive:*<br>*- incentive phase:* No	No incentives: Weight loss goal & educational materials	Physiological outcome	Passive	8 weeks	-	Absolute weight loss in kilograms & objectively verified	*Incentive phase*<br>Control: M = -1.19, SE = .11<br>Incentive: M = -.20, SE = .11
van der Swaluw (2018) **AB**	N = 163<br>*Control:* 48<br>*Incentive:* 115<br>(**A:** 60; **B:** 55)<br>overweight adults	**A**: *Form:* Lottery<br>*Magnitude:* $1300<br>*Certainty:* Uncertain chance<br>*Participation incentive*<br>*- incentive phase:* Yes<br>*- post-incentive phase:* Yes<br><br>**B**: *Form:* Lottery<br>*Magnitude:* $1300 and family vacation voucher<br>*Certainty:* Uncertain chance<br>*Participation incentive*<br>*- incentive phase:* Yes<br>*- post-incentive phase:* Yes	No incentives: Exercise goal, monthly progress & gym supervision	Process behavior	Passive	13 weeks	39 weeks	Absolute weight loss in kilograms & objectively verified<br><br>(compared to previous measurement)	**A:** *Incentive phase*<br>Control: M = -1.02, SE = 5.47<br>Incentive: M = +.90 SE = 1.85<br><br>*Post-incentive phase*<br>Control: M = -1.33, SE = 6.79<br>Incentive: M = -.63, SE = 2.08<br><br>**B:** *Incentive phase*<br>Control: M = -1.02, SE = 5.47<br>Incentive: M = +1.11, SE = 1.94<br><br>*Post-incentive phase*<br>Control: M = -1.33, SE = 6.79<br>Incentive: M = +.44, SE = 2.13
VanEpps (2019) **ABC**	N = 708<br>*Control:* 170	**A**: *Form:* Cash<br>*Magnitude:* $240<br>*Certainty:* Certain	No incentives: Diabetes	**A:** Physiological outcomes	Interactive	16 weeks	-	Absolute weight loss in pounds &	**A:** *Incentive phase*<br>Control: M = -7.9, SE = 1.63

	Incentive: 533<br>(**A:** 181; **B:** 182; **C:** 170) adults with pre-diabetes	*Participation incentive - incentive phase:* Yes<br><br>**B**: *Form:* Cash<br>*Magnitude:* $240<br>*Certainty:* Certain<br>*Participation incentive: - incentive phase:* Yes<br><br>**C**: *Form:* Cash<br>*Magnitude:* $240<br>*Certainty:* Certain<br>*Participation incentive - incentive phase:* Yes	prevention program (weekly behavior change sessions)	 **B:** Process behavior<br><br>**C:** Multiple - Process behavior & physiologic al outcomes				objectively verified	Incentive: M = -6.6, SE = 1.27<br><br>**B:** *Incentive phase*<br>Control: M = -7.9, SE = 1.63<br>Incentive: M = -7.3, SE = 1.12<br><br>**C:** *Incentive phase*<br>Control: M = -7.9, SE = 1.63<br>Incentive: M = -5.8, SE = 1.53
Voils (2021)<br>**ABC**	N = 93<br>*Control:* 19<br>*Incentive:* 58<br>(**A:** 20; **B:** 19; **C:** 19) obese adults	**A**: *Form:* Cash<br>*Magnitude:* $300<br>*Certainty:* Certain<br>*Participation incentive: - incentive phase:* Yes<br><br>**B**: *Form:* Cash<br>*Magnitude:* $300<br>*Certainty:* Certain<br>*Participation incentive - incentive phase:* Yes<br><br>**C:** *Form:* Cash<br>*Magnitude:* $300<br>*Certainty:* Certain<br>*Participation incentive - incentive phase:* Yes	No incentives: Group-based weight loss program	**A:** Multiple - Physiologic al outcomes & process behavior<br><br>**B:** Process behavior<br><br>**C:** Physiologic al outcome	Interacti ve	24 weeks	-	Absolute weight loss in pounds & objectively verified	**A:** *Incentive phase*<br>Control: M = -10.02, SE = 3.78<br>Incentive: M = -19.43, SE = 2.13<br><br>**B:** *Incentive phase*<br>Control: M = -10.02, SE = 3.78<br>Incentive: M = -16.01, SE = 3.41<br><br>**C:** Incentive phase<br>Control: M = -10.02, SE = 3.78<br>Incentive: M = -12.34, SE = 4.51

Volpp (2008) **AB**	N = 57<br>*Control:* 19<br>*Incentive:* 38 (**A:** 19; **B:** 19)<br>obese adults	**A**: *Form:* Deposit contract<br>*Magnitude:* $1104.25<br>*Certainty:* certain<br>*Participation incentive*<br>*- incentive phase:* Yes<br>*- post-incentive phase:* Unknown<br><br>**B**: *Form*: Lottery<br>*Magnitude:* $386<br>*Certainty:* certain chance<br>*Participation incentive*<br>*- incentive phase*: Yes<br>*- post-incentive phase:* Unknown	No incentive: Free weight scale, consultation with dietitian & weight-monitoring program	Physiological outcome	Interactive	16 weeks	31.5 weeks	Absolute weight loss in pounds & objectively verified	**A:** *Incentive phase*<br>Control: M = -3.90, SE = 2.09<br>Incentive: M = -14.0, SE = 2.34<br><br>*Post-incentive phase*<br>Control: M = -4.4, SE = 2.42<br>Incentive: M = -6.2, SE = 2.77<br><br>**B:** *Incentive phase*<br>Control: M = -3.90, SE = 2.09<br>Incentive: M = -13.1 SE = 3.4<br><br>*Post-incentive phase*<br>Control: M = -4.4, SE = 2.4<br>Incentive: M = -9.2, SE = 4.71
West (2020)	N = 418<br>*Control:* 212<br>*Incentive:* 206<br>obese adults	*Form:* (Voucher/coupon for) free service(s) or good(s)<br>*Magnitude:* $230<br>*Certainty:* Certain<br>*Participation incentive*<br>*- incentive phase:* Unknown	No incentives: Group-based behavioral weight management program	Multiple - Process behavior & physiological outcome	Interactive	27 weeks	-	Absolute weight loss in kilograms & objectively verified	*Incentive phase*<br>Control: M = -4.7, SE = 4.34<br>Incentive: M = -6.4, SE = .38

Wing (1996)	N = 35<br>*Control:* 15<br>*Incentive:* 20 obese adults	*Form:* Lottery<br>*Magnitude:* $5550<br>*Certainty:* Uncertain chance<br>*Participation incentive - incentive phase:* No	No incentive: Behavioral weight control program	Process behavior	Interactive	24 weeks	-	Absolute weight loss in kilograms & objectively verified	*Incentive phase*<br>Control: M = -6.7, SE = 1.91<br>Incentive: M = -5.9, SE = 1.50

Note. *Total N:* The sample size reported is based on the reported intervention or post-intervention sample sizes (not the bassline sample sizes) to account for dropout. *Incentive characteristics*: Magnitude is the total possible incentive earnings in $ received for the health behaviors or outcomes related to weight loss, regardless of the certainty of receiving these incentives (the magnitude was converted into US$ at the date of publication of the specific article); Frequency/immediacy delivery is interpreted as the frequency/immediacy of incentive delivery when it is salient/noticeable for the recipient; Participation incentive is when participants receive an incentive for participating, coming to an assessment etc.; *Results:* When there is a minus (-) in front of the amount of weight loss for a certain condition, it means that there was weight loss from baseline till that time-point and a plus (+) indicates weight gain.
*Baseline data included to show change in weight for the timepoint studies

Appendix B

Additional forest plots

Study name	Time point	Statistics for each study							Weight (Random)	
		Std diff in means	Standard error	Variance	Lower limit	Upper limit	Z-Value	p-Value	Relative weight	Relative weight
Desai (2020)	16 weeks	-0,163	0,087	0,008	-0,333	0,007	-1,877	0,060	3,73	
Dombrowski (2020)	54 weeks	0,332	0,243	0,059	-0,144	0,807	1,368	0,171	2,06	
Faghri (2014) B	16 weeks	0,559	0,346	0,120	-0,119	1,237	1,616	0,106	1,35	
Faghri (2014) A	16 weeks	0,563	0,331	0,109	-0,086	1,211	1,701	0,089	1,43	
Finkelstein (2016)	27 weeks	0,046	0,100	0,010	-0,150	0,242	0,457	0,648	3,59	
Finkelstein (2017) A	36 weeks	0,265	0,229	0,052	-0,184	0,713	1,158	0,247	2,18	
Finkelstein (2017) B	36 weeks	0,503	0,253	0,064	0,007	0,998	1,988	0,047	1,97	
Geliebter (2013)	8 weeks	0,047	0,297	0,088	-0,535	0,630	0,159	0,873	1,64	
Glanz (2021) A	81 weeks	-0,096	0,153	0,023	-0,395	0,203	-0,630	0,528	2,99	
Glanz (2021) B	81 weeks	-0,167	0,153	0,023	-0,466	0,133	-1,092	0,275	2,98	
Jeffery (1998) A	81 weeks	0,102	0,261	0,068	-0,409	0,614	0,393	0,695	1,91	
Jeffery (1998) B	81 weeks	0,349	0,261	0,068	-0,162	0,860	1,337	0,181	1,91	
John (2011)	32 weeks	0,494	0,306	0,094	-0,106	1,094	1,614	0,107	1,58	
Kranker (2018) A	40,5 weeks	0,240	0,336	0,113	-0,418	0,897	0,714	0,475	1,40	
Kranker (2018) B	40,5 weeks	0,229	0,583	0,340	-0,914	1,372	0,393	0,694	0,60	
Kranker (2018) C	40,5 weeks	0,263	0,539	0,290	-0,793	1,319	0,488	0,625	0,68	
Kullgren (2013)	24 weeks	0,234	0,240	0,058	-0,236	0,704	0,974	0,330	2,08	
Kullgren (2016) A	24 weeks	0,617	0,354	0,126	-0,077	1,312	1,742	0,082	1,30	
Kullgren (2016) B	24 weeks	0,659	0,355	0,126	-0,037	1,355	1,855	0,064	1,30	
Kullgren (2016) C	24 weeks	0,354	0,350	0,123	-0,332	1,040	1,011	0,312	1,32	
Ladapo (2023) A	27 weeks	0,402	0,117	0,014	0,172	0,632	3,423	0,001	3,39	
Ladapo (2023) B	27 weeks	0,455	0,117	0,014	0,224	0,685	3,869	0,000	3,39	
Leahey (2015)	13,5 weeks	0,316	0,150	0,023	0,022	0,610	2,105	0,035	3,02	
Memon (2018)	5 weeks	0,034	0,267	0,071	-0,490	0,558	0,127	0,899	1,86	
Patel (2016) A	54 weeks	0,104	0,284	0,080	-0,452	0,660	0,365	0,715	1,73	
Patel (2016) B	54 weeks	0,116	0,284	0,081	-0,442	0,673	0,407	0,684	1,73	
Patel (2016) C	54 weeks	0,113	0,283	0,080	-0,442	0,667	0,398	0,690	1,74	
Petry (2011)	12 weeks	0,554	0,272	0,074	0,021	1,088	2,035	0,042	1,82	
Pope (2014)	12 weeks	0,030	0,231	0,053	-0,423	0,483	0,129	0,897	2,16	
Pratt (2023) A	54 weeks	0,041	0,201	0,040	-0,353	0,435	0,205	0,838	2,45	
Pratt (2023) B	54 weeks	0,041	0,112	0,012	-0,177	0,260	0,372	0,710	3,46	
Pratt (2023) C	54 weeks	0,095	0,300	0,090	-0,494	0,683	0,316	0,752	1,62	
Pratt (2023) D	54 weeks	-0,035	0,113	0,013	-0,258	0,187	-0,312	0,755	3,44	
Rounds (2020)	12 weeks	-0,105	0,199	0,039	-0,494	0,284	-0,529	0,597	2,48	
Saccone (1978) A	9 weeks	0,037	0,677	0,458	-1,290	1,364	0,055	0,956	0,46	
Saccone (1978) B	9 weeks	0,589	0,639	0,409	-0,664	1,842	0,921	0,357	0,51	
Saccone (1978) C	9 weeks	0,315	0,680	0,463	-1,019	1,648	0,462	0,644	0,46	
Saccone (1978) D	9 weeks	1,472	0,701	0,491	0,098	2,846	2,100	0,036	0,43	
Shin (2017)	12 weeks	0,604	0,252	0,063	0,110	1,098	2,399	0,016	1,98	
Sykes-Muskett (2017)	8 weeks	-2,029	0,385	0,148	-2,784	-1,274	-5,269	0,000	1,16	
van der Swaluw (2018) A	13 weeks	-0,082	0,242	0,058	-0,555	0,392	-0,339	0,735	2,07	
van der Swaluw (2018) B	13 weeks	-0,089	0,245	0,060	-0,569	0,391	-0,363	0,716	2,04	
VanEpps (2019) A	16 weeks	-0,071	0,152	0,023	-0,370	0,227	-0,469	0,639	2,99	
VanEpps (2019) B	16 weeks	-0,036	0,152	0,023	-0,334	0,262	-0,235	0,814	2,99	
VanEpps (2019) C	16 weeks	-0,103	0,153	0,024	-0,404	0,197	-0,674	0,500	2,98	
Voils (2021) A	24 weeks	1,125	0,482	0,232	0,181	2,069	2,336	0,020	0,82	
Voils (2021) B	24 weeks	0,404	0,462	0,214	-0,503	1,310	0,873	0,383	0,88	
Voils (2021) C	24 weeks	0,124	0,459	0,211	-0,776	1,024	0,271	0,787	0,89	
Volpp (2008) A	16 weeks	1,024	0,420	0,176	0,201	1,847	2,439	0,015	1,02	
Volpp (2008) B	16 weeks	0,793	0,411	0,169	-0,012	1,599	1,930	0,054	1,05	
West (2020)	27 weeks	0,439	0,099	0,010	0,245	0,633	4,433	0,000	3,60	
Wing (1996)	24 weeks	-0,114	0,342	0,117	-0,784	0,556	-0,334	0,738	1,37	
		0,168	0,049	0,002	0,073	0,263	3,457	0,001		

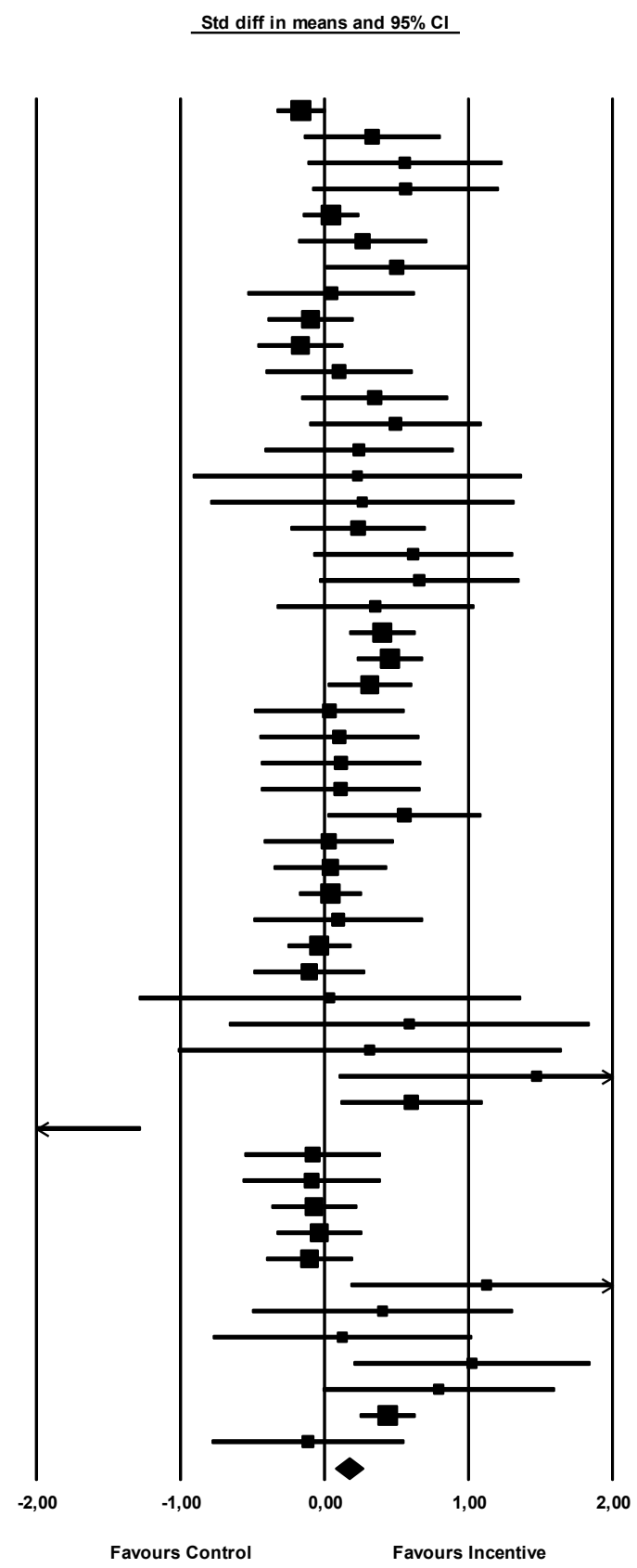

Figure B1. Overall analysis for the incentive phase including all studies.

Group by Target	Study name	Time point	Std diff in means	Standard error	Variance	Lower limit	Upper limit	Z-Value	p-Value	Relative weight
			Statistics for each study							
Life	Finkelstein (2016)	27 weeks	0,046	0,100	0,010	-0,150	0,242	0,457	0,648	64,26
Life	Kranker (2018) B	40,5 weeks	0,229	0,583	0,340	-0,914	1,372	0,393	0,694	5,55
Life	Memon (2018)	5 weeks	0,034	0,267	0,071	-0,490	0,558	0,127	0,899	21,63
Life	Saccone (1978) B	9 weeks	0,589	0,639	0,409	-0,664	1,842	0,921	0,357	4,66
Life	Saccone (1978) D	9 weeks	1,472	0,701	0,491	0,098	2,846	2,100	0,036	3,91
Life			0,134	0,142	0,020	-0,143	0,412	0,950	0,342	
Multi	Desai (2020)	16 weeks	-0,163	0,087	0,008	-0,333	0,007	-1,877	0,060	14,48
Multi	Finkelstein (2017) A	36 weeks	0,265	0,229	0,052	-0,184	0,713	1,158	0,247	9,97
Multi	Finkelstein (2017) B	36 weeks	0,503	0,253	0,064	0,007	0,998	1,988	0,047	9,22
Multi	Kranker (2018) C	40,5 weeks	0,263	0,539	0,290	-0,793	1,319	0,488	0,625	3,75
Multi	Ladapo (2023) A	27 weeks	0,402	0,117	0,014	0,172	0,632	3,423	0,001	13,62
Multi	Petry (2011)	12 weeks	0,554	0,272	0,074	0,021	1,088	2,035	0,042	8,65
Multi	Shin (2017)	12 weeks	0,604	0,252	0,063	0,110	1,098	2,399	0,016	9,25
Multi	VanEpps (2019) C	16 weeks	-0,103	0,153	0,024	-0,404	0,197	-0,674	0,500	12,46
Multi	Voils (2021) A	24 weeks	1,125	0,482	0,232	0,181	2,069	2,336	0,020	4,43
Multi	West (2020)	27 weeks	0,439	0,099	0,010	0,245	0,633	4,433	0,000	14,16
Multi			0,317	0,120	0,014	0,082	0,551	2,647	0,008	
Phys	Dombrowski (2020)	54 weeks	0,332	0,243	0,059	-0,144	0,807	1,368	0,171	4,91
Phys	Faghri (2014) A	16 weeks	0,563	0,331	0,109	-0,086	1,211	1,701	0,089	3,12
Phys	Faghri (2014) B	16 weeks	0,559	0,346	0,120	-0,119	1,237	1,616	0,106	2,91
Phys	Glanz (2021) A	81 weeks	-0,096	0,153	0,023	-0,395	0,203	-0,630	0,528	8,22
Phys	Glanz (2021) B	81 weeks	-0,167	0,153	0,023	-0,466	0,133	-1,092	0,275	8,21
Phys	John (2011)	32 weeks	0,494	0,306	0,094	-0,106	1,094	1,614	0,107	3,52
Phys	Kranker (2018) A	40,5 weeks	0,240	0,336	0,113	-0,418	0,897	0,714	0,475	3,06
Phys	Kullgren (2013)	24 weeks	0,234	0,240	0,058	-0,236	0,704	0,974	0,330	4,99
Phys	Kullgren (2016) A	24 weeks	0,617	0,354	0,126	-0,077	1,312	1,742	0,082	2,80
Phys	Kullgren (2016) B	24 weeks	0,659	0,355	0,126	-0,037	1,355	1,855	0,064	2,79
Phys	Kullgren (2016) C	24 weeks	0,354	0,350	0,123	-0,332	1,040	1,011	0,312	2,85
Phys	Ladapo (2023) B	27 weeks	0,455	0,117	0,014	0,224	0,685	3,869	0,000	10,02
Phys	Leahey (2015)	13,5 weeks	0,316	0,150	0,023	0,022	0,610	2,105	0,035	8,34
Phys	Patel (2016) A	54 weeks	0,104	0,284	0,080	-0,452	0,660	0,365	0,715	3,95
Phys	Patel (2016) B	54 weeks	0,116	0,284	0,081	-0,442	0,673	0,407	0,684	3,93
Phys	Patel (2016) C	54 weeks	0,113	0,283	0,080	-0,442	0,667	0,398	0,690	3,96
Phys	Rounds (2020)	12 weeks	-0,105	0,199	0,039	-0,494	0,284	-0,529	0,597	6,30
Phys	Saccone (1978) A	9 weeks	0,037	0,677	0,458	-1,290	1,364	0,055	0,956	0,89
Phys	Saccone (1978) C	9 weeks	0,315	0,680	0,463	-1,019	1,648	0,462	0,644	0,88
Phys	VanEpps (2019) A	16 weeks	-0,071	0,152	0,023	-0,370	0,227	-0,469	0,639	8,24
Phys	Voils (2021) C	24 weeks	0,124	0,459	0,211	-0,776	1,024	0,271	0,787	1,81
Phys	Volpp (2008) A	16 weeks	1,024	0,420	0,176	0,201	1,847	2,439	0,015	2,11
Phys	Volpp (2008) B	16 weeks	0,793	0,411	0,169	-0,012	1,599	1,930	0,054	2,19
Phys			0,228	0,066	0,004	0,098	0,357	3,453	0,001	
Proc	Geliebter (2013)	8 weeks	0,047	0,297	0,088	-0,535	0,630	0,159	0,873	3,29
Proc	Jeffery (1998) A	81 weeks	0,102	0,261	0,068	-0,409	0,614	0,393	0,695	4,28
Proc	Jeffery (1998) B	81 weeks	0,349	0,261	0,068	-0,162	0,860	1,337	0,181	4,28
Proc	Pope (2014)	12 weeks	0,030	0,231	0,053	-0,423	0,483	0,129	0,897	5,46
Proc	Pratt (2023) A	54 weeks	0,041	0,201	0,040	-0,353	0,435	0,205	0,838	7,20
Proc	Pratt (2023) B	54 weeks	0,041	0,112	0,012	-0,177	0,260	0,372	0,710	23,36
Proc	Pratt (2023) C	54 weeks	0,095	0,300	0,090	-0,494	0,683	0,316	0,752	3,23
Proc	Pratt (2023) D	54 weeks	-0,035	0,113	0,013	-0,258	0,187	-0,312	0,755	22,63
Proc	van der Swaluw (2018) A	13 weeks	-0,082	0,242	0,058	-0,555	0,392	-0,339	0,735	4,99
Proc	van der Swaluw (2018) B	13 weeks	-0,089	0,245	0,060	-0,569	0,391	-0,363	0,716	4,86
Proc	VanEpps (2019) B	16 weeks	-0,036	0,152	0,023	-0,334	0,262	-0,235	0,814	12,58
Proc	Voils (2021) B	24 weeks	0,404	0,462	0,214	-0,503	1,310	0,873	0,383	1,36
Proc	Wing (1996)	24 weeks	-0,114	0,342	0,117	-0,784	0,556	-0,334	0,738	2,49
Proc			0,020	0,054	0,003	-0,086	0,126	0,370	0,712	
Overall			0,127	0,038	0,001	0,053	0,201	3,345	0,001	

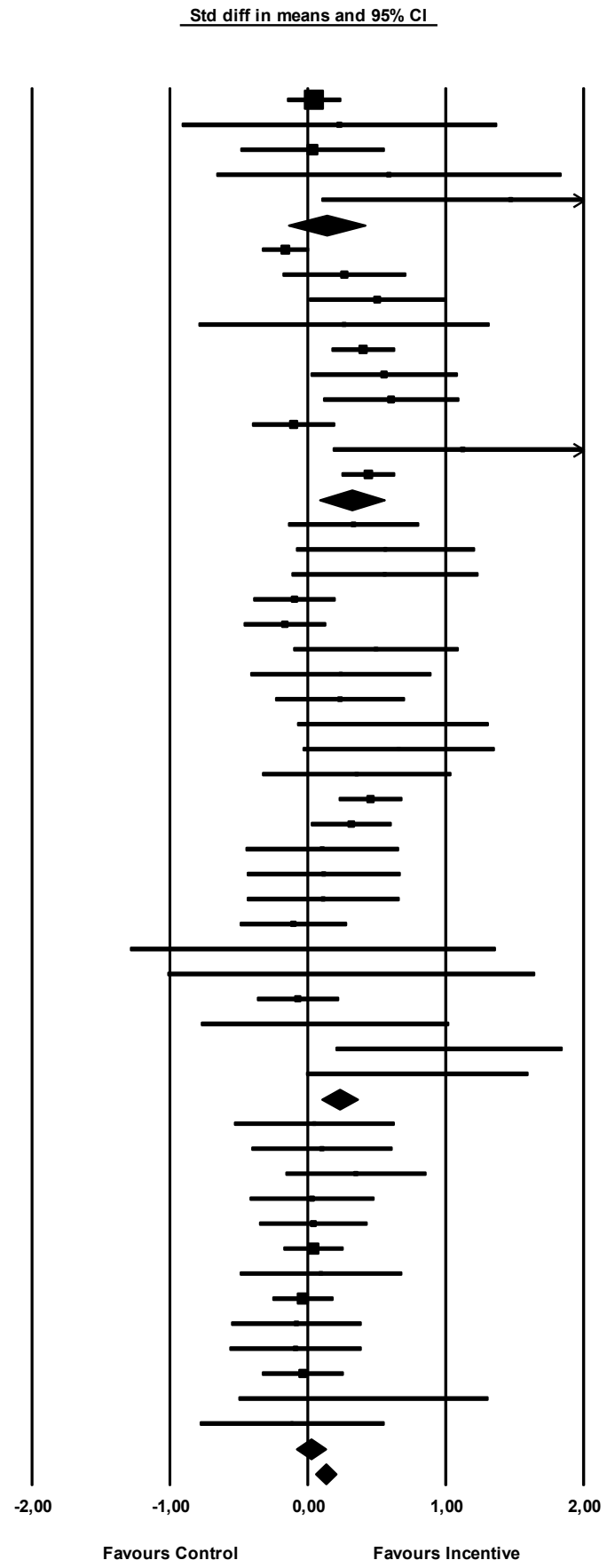

Figure B2. Moderator analysis for incentive target for the incentive phase.

Group by Target	Studyname	Time point	Std diff in means	Standard error	Variance	Lower limit	Upper limit	Z-Value	p-Value	Relative weight
			Statistics for each study							
Life	Finkelstein (2016)	27 weeks	0,032	0,100	0,010	-0,164	0,228	0,316	0,752	97,33
Life	Kranker (2018) B	67.5 weeks	0,288	0,604	0,364	-0,895	1,470	0,476	0,634	2,67
Life			0,038	0,099	0,010	-0,155	0,232	0,390	0,697	
Multi	Desai (2020)	36 weeks	-0,124	0,145	0,021	-0,409	0,161	-0,854	0,393	29,70
Multi	Finkelstein (2017) A	4 weeks	0,017	0,228	0,052	-0,430	0,464	0,076	0,940	12,10
Multi	Finkelstein (2017) B	4 weeks	0,000	0,249	0,062	-0,488	0,488	0,000	1,000	10,13
Multi	Kranker (2018) C	67.5 weeks	-0,478	0,608	0,370	-1,671	0,714	-0,787	0,431	1,70
Multi	Ladapo (2023) A	54 weeks	0,052	0,116	0,014	-0,176	0,281	0,450	0,653	46,37
Multi			-0,019	0,079	0,006	-0,174	0,137	-0,235	0,814	
Phys	Glanz (2021) A	27 weeks	-0,010	0,152	0,023	-0,309	0,289	-0,068	0,946	14,73
Phys	Glanz (2021) B	27 weeks	-0,002	0,152	0,023	-0,300	0,297	-0,010	0,992	14,73
Phys	Kranker (2018) A	67.5 weeks	0,240	0,380	0,144	-0,504	0,984	0,632	0,527	2,38
Phys	Kullgren (2013)	12 weeks	0,112	0,239	0,057	-0,357	0,581	0,468	0,640	5,98
Phys	Kullgren (2016) A	12 weeks	0,141	0,348	0,121	-0,542	0,824	0,403	0,687	2,82
Phys	Kullgren (2016) B	12 weeks	0,047	0,348	0,121	-0,636	0,729	0,134	0,893	2,82
Phys	Kullgren (2016) C	12 weeks	0,125	0,348	0,121	-0,558	0,807	0,357	0,721	2,82
Phys	Ladapo (2023) B	54 weeks	0,085	0,116	0,014	-0,142	0,313	0,735	0,462	25,36
Phys	Leahey (2015)	40,5 weeks	0,023	0,149	0,022	-0,269	0,316	0,157	0,876	15,41
Phys	Rounds (2020)	12 weeks	-0,019	0,198	0,039	-0,408	0,370	-0,096	0,923	8,70
Phys	Volpp (2008) A	31,5 weeks	0,545	0,404	0,163	-0,246	1,337	1,350	0,177	2,10
Phys	Volpp (2008) B	31,5 weeks	0,245	0,399	0,159	-0,536	1,027	0,616	0,538	2,15
Phys			0,060	0,059	0,003	-0,055	0,174	1,022	0,307	
Proc	Geliebter (2013)	4 weeks	0,050	0,324	0,105	-0,586	0,686	0,154	0,878	15,29
Proc	Pope (2014)	20 weeks	0,010	0,231	0,053	-0,443	0,463	0,043	0,965	30,19
Proc	van der Swaluw (2018) A	52 weeks after baseline	0,026	0,242	0,058	-0,447	0,500	0,110	0,913	27,61
Proc	van der Swaluw (2018) B	52 weeks after baseline	-0,004	0,245	0,060	-0,484	0,475	-0,018	0,986	26,91
Proc			0,017	0,127	0,016	-0,232	0,266	0,133	0,894	
Overall			0,032	0,040	0,002	-0,047	0,111	0,785	0,432	

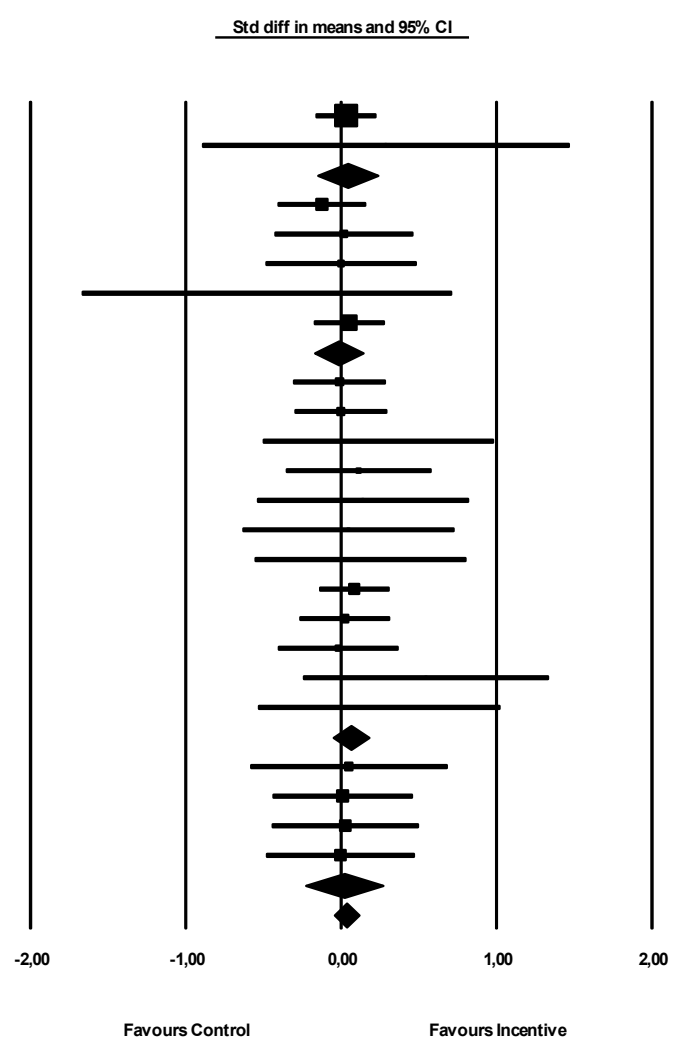

Figure B3. Moderator analysis for incentive target for the post-incentive phase.

Group by BCT_Type	Studyname		Std diff in means	Standard error	Variance	Lower limit	Upper limit	Z-Value	p-Value	Relative weight
Interactive	Desai (2020)	16 weeks	-0,163	0,087	0,008	-0,333	0,007	-1,877	0,060	5,56
Interactive	Faghri (2014) A	16 weeks	0,563	0,331	0,109	-0,086	1,211	1,701	0,089	1,98
Interactive	Faghri (2014) B	16 weeks	0,559	0,346	0,120	-0,119	1,237	1,616	0,106	1,86
Interactive	Finkelstein (2017) A	36 weeks	0,265	0,229	0,052	-0,184	0,713	1,158	0,247	3,09
Interactive	Finkelstein (2017) B	36 weeks	0,503	0,253	0,064	0,007	0,998	1,988	0,047	2,78
Interactive	Glanz (2021) A	81 weeks	-0,096	0,153	0,023	-0,395	0,203	-0,630	0,528	4,35
Interactive	Glanz (2021) B	81 weeks	-0,167	0,153	0,023	-0,466	0,133	-1,092	0,275	4,34
Interactive	Jeffery (1998) A	81 weeks	0,102	0,261	0,068	-0,409	0,614	0,393	0,695	2,68
Interactive	Jeffery (1998) B	81 weeks	0,349	0,261	0,068	-0,162	0,860	1,337	0,181	2,68
Interactive	John (2011)	32 weeks	0,494	0,306	0,094	-0,106	1,094	1,614	0,107	2,20
Interactive	Kranker (2018) A	40,5 weeks	0,240	0,336	0,113	-0,418	0,897	0,714	0,475	1,94
Interactive	Kranker (2018) B	40,5 weeks	0,229	0,583	0,340	-0,914	1,372	0,393	0,694	0,80
Interactive	Kranker (2018) C	40,5 weeks	0,263	0,539	0,290	-0,793	1,319	0,488	0,625	0,92
Interactive	Ladapo (2023) A	27 weeks	0,402	0,117	0,014	0,172	0,632	3,423	0,001	5,01
Interactive	Ladapo (2023) B	27 weeks	0,455	0,117	0,014	0,224	0,685	3,869	0,000	5,01
Interactive	Leahey (2015)	13,5 weeks	0,316	0,150	0,023	0,022	0,610	2,105	0,035	4,39
Interactive	Petry (2011)	12 weeks	0,554	0,272	0,074	0,021	1,088	2,035	0,042	2,54
Interactive	Pratt (2023) B	54 weeks	0,041	0,112	0,012	-0,177	0,260	0,372	0,710	5,12
Interactive	Pratt (2023) C	54 weeks	0,095	0,300	0,090	-0,494	0,683	0,316	0,752	2,25
Interactive	Pratt (2023) D	54 weeks	-0,035	0,113	0,013	-0,258	0,187	-0,312	0,755	5,08
Interactive	Rounds (2020)	12 weeks	-0,105	0,199	0,039	-0,494	0,284	-0,529	0,597	3,55
Interactive	Saccone (1978) A	9 weeks	0,037	0,677	0,458	-1,290	1,364	0,055	0,956	0,62
Interactive	Saccone (1978) B	9 weeks	0,589	0,639	0,409	-0,664	1,842	0,921	0,357	0,68
Interactive	Saccone (1978) C	9 weeks	0,315	0,680	0,463	-1,019	1,648	0,462	0,644	0,61
Interactive	Saccone (1978) D	9 weeks	1,472	0,701	0,491	0,098	2,846	2,100	0,036	0,58
Interactive	Shin (2017)	12 weeks	0,604	0,252	0,063	0,110	1,098	2,399	0,016	2,79
Interactive	VanEpps (2019) A	16 weeks	-0,071	0,152	0,023	-0,370	0,227	-0,469	0,639	4,35
Interactive	VanEpps (2019) B	16 weeks	-0,036	0,152	0,023	-0,334	0,262	-0,235	0,814	4,35
Interactive	VanEpps (2019) C	16 weeks	-0,103	0,153	0,024	-0,404	0,197	-0,674	0,500	4,33
Interactive	Voils (2021) A	24 weeks	1,125	0,482	0,232	0,181	2,069	2,336	0,020	1,11
Interactive	Voils (2021) B	24 weeks	0,404	0,462	0,214	-0,503	1,310	0,873	0,383	1,19
Interactive	Voils (2021) C	24 weeks	0,124	0,459	0,211	-0,776	1,024	0,271	0,787	1,20
Interactive	Volpp (2008) A	16 weeks	1,024	0,420	0,176	0,201	1,847	2,439	0,015	1,39
Interactive	Volpp (2008) B	16 weeks	0,793	0,411	0,169	-0,012	1,599	1,930	0,054	1,44
Interactive	West (2020)	27 weeks	0,439	0,099	0,010	0,245	0,633	4,433	0,000	5,35
Interactive	Wing (1996)	24 weeks	-0,114	0,342	0,117	-0,784	0,556	-0,334	0,738	1,89
Interactive			0,213	0,056	0,003	0,103	0,322	3,807	0,000	
Passive	Dombrowski (2020)	54 weeks	0,332	0,243	0,059	-0,144	0,807	1,368	0,171	5,69
Passive	Finkelstein (2016)	27 weeks	0,046	0,100	0,010	-0,150	0,242	0,457	0,648	33,51
Passive	Geliebter (2013)	8 weeks	0,047	0,297	0,088	-0,535	0,630	0,159	0,873	3,79
Passive	Kullgren (2013)	24 weeks	0,234	0,240	0,058	-0,236	0,704	0,974	0,330	5,83
Passive	Kullgren (2016) A	24 weeks	0,617	0,354	0,126	-0,077	1,312	1,742	0,082	2,67
Passive	Kullgren (2016) B	24 weeks	0,659	0,355	0,126	-0,037	1,355	1,855	0,064	2,66
Passive	Kullgren (2016) C	24 weeks	0,354	0,350	0,123	-0,332	1,040	1,011	0,312	2,73
Passive	Memon (2018)	5 weeks	0,034	0,267	0,071	-0,490	0,558	0,127	0,899	4,69
Passive	Patel (2016) A	54 weeks	0,104	0,284	0,080	-0,452	0,660	0,365	0,715	4,17
Passive	Patel (2016) B	54 weeks	0,116	0,284	0,081	-0,442	0,673	0,407	0,684	4,14
Passive	Patel (2016) C	54 weeks	0,113	0,283	0,080	-0,442	0,667	0,398	0,690	4,19
Passive	Pope (2014)	12 weeks	0,030	0,231	0,053	-0,423	0,483	0,129	0,897	6,29
Passive	Pratt (2023) A	54 weeks	0,041	0,201	0,040	-0,353	0,435	0,205	0,838	8,29
Passive	van der Swaluw (2018) A	13 weeks	-0,082	0,242	0,058	-0,555	0,392	-0,339	0,735	5,74
Passive	van der Swaluw (2018) B	13 weeks	-0,089	0,245	0,060	-0,569	0,391	-0,363	0,716	5,60
Passive			0,104	0,058	0,003	-0,009	0,218	1,802	0,072	
Overall			0,160	0,040	0,002	0,082	0,239	3,990	0,000	

(Statistics for each study)

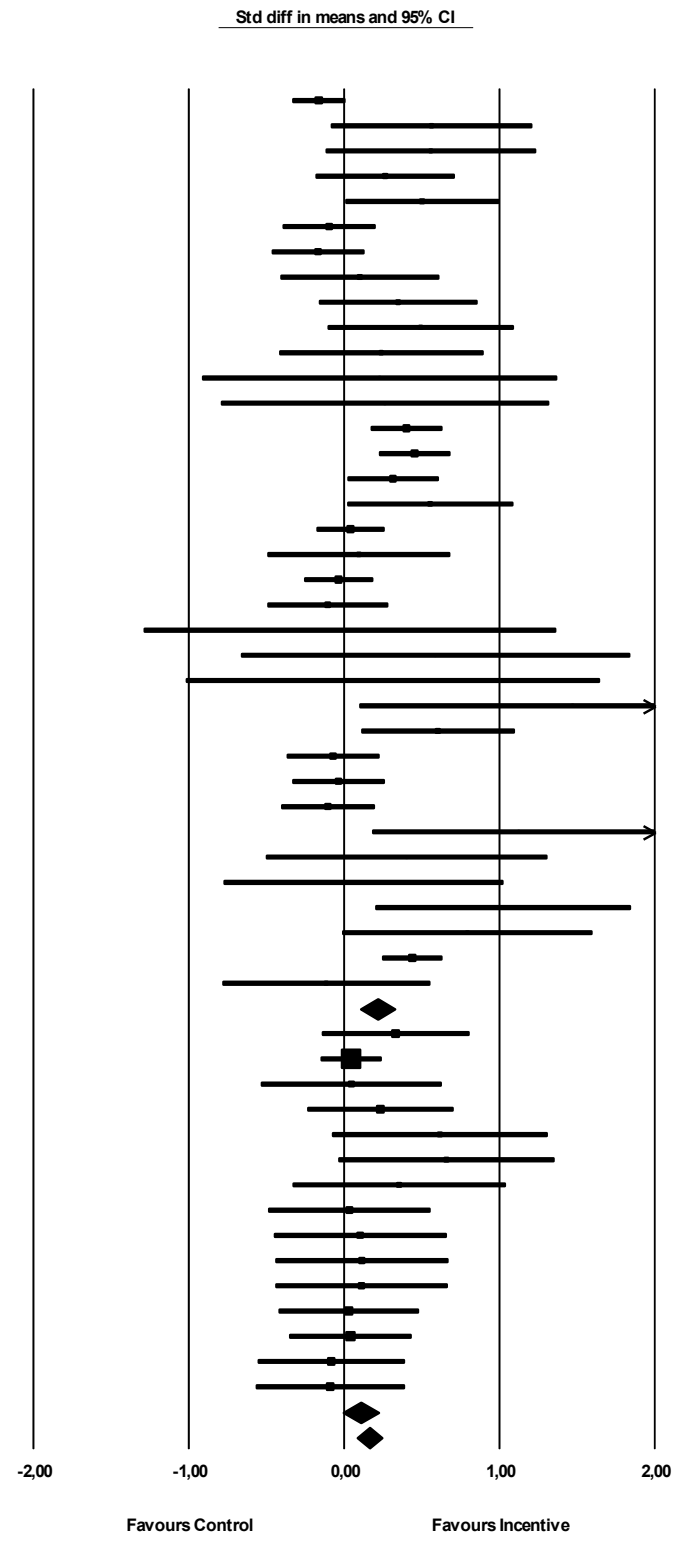

Figure B4. Moderator analysis for passive or interactive BCTs for the incentive phase.

Group by BCT_type	Studyname	Time point	Std diff in means	Standard error	Variance	Lower limit	Upper limit	Z-Value	p-Value	Relative weight
Interactive	Desai (2020)	36 weeks	-0,124	0,145	0,021	-0,409	0,161	-0,854	0,393	11,48
Interactive	Finkelstein (2017) A	4 weeks	0,017	0,228	0,052	-0,430	0,464	0,076	0,940	4,68
Interactive	Finkelstein (2017) B	4 weeks	0,000	0,249	0,062	-0,488	0,488	0,000	1,000	3,92
Interactive	Glanz (2021) A	27 weeks	-0,010	0,152	0,023	-0,309	0,289	-0,068	0,946	10,45
Interactive	Glanz (2021) B	27 weeks	-0,002	0,152	0,023	-0,300	0,297	-0,010	0,992	10,45
Interactive	Kranker (2018) A	67.5 weeks	0,240	0,380	0,144	-0,504	0,984	0,632	0,527	1,69
Interactive	Kranker (2018) B	67.5 weeks	0,288	0,604	0,364	-0,895	1,470	0,476	0,634	0,67
Interactive	Kranker (2018) C	67.5 weeks	-0,478	0,608	0,370	-1,671	0,714	-0,787	0,431	0,66
Interactive	Ladapo (2023) A	54 weeks	0,052	0,116	0,014	-0,176	0,281	0,450	0,653	17,92
Interactive	Ladapo (2023) B	54 weeks	0,085	0,116	0,014	-0,142	0,313	0,735	0,462	17,99
Interactive	Leahey (2015)	40,5 weeks	0,023	0,149	0,022	-0,269	0,316	0,157	0,876	10,93
Interactive	Rounds (2020)	12 weeks	-0,019	0,198	0,039	-0,408	0,370	-0,096	0,923	6,17
Interactive	Volpp (2008) A	31,5 weeks	0,545	0,404	0,163	-0,246	1,337	1,350	0,177	1,49
Interactive	Volpp (2008) B	31,5 weeks	0,245	0,399	0,159	-0,536	1,027	0,616	0,538	1,53
Interactive			0,026	0,049	0,002	-0,070	0,123	0,530	0,596	
Passive	Finkelstein (2016)	27 weeks	0,032	0,100	0,010	-0,164	0,228	0,316	0,752	48,94
Passive	Geliebter (2013)	4 weeks	0,050	0,324	0,105	-0,586	0,686	0,154	0,878	4,65
Passive	Kullgren (2013)	12 weeks	0,112	0,239	0,057	-0,357	0,581	0,468	0,640	8,55
Passive	Kullgren (2016) A	12 weeks	0,141	0,348	0,121	-0,542	0,824	0,403	0,687	4,03
Passive	Kullgren (2016) B	12 weeks	0,047	0,348	0,121	-0,636	0,729	0,134	0,893	4,04
Passive	Kullgren (2016) C	12 weeks	0,125	0,348	0,121	-0,558	0,807	0,357	0,721	4,03
Passive	Pope (2014)	20 weeks	0,010	0,231	0,053	-0,443	0,463	0,043	0,965	9,18
Passive	van der Swaluw (2018) A	52 weeks after baseline	0,026	0,242	0,058	-0,447	0,500	0,110	0,913	8,39
Passive	van der Swaluw (2018) B	52 weeks after baseline	-0,004	0,245	0,060	-0,484	0,475	-0,018	0,986	8,18
Passive			0,043	0,070	0,005	-0,094	0,180	0,611	0,541	
Overall			0,032	0,040	0,002	-0,047	0,111	0,785	0,432	

(Statistics for each study)

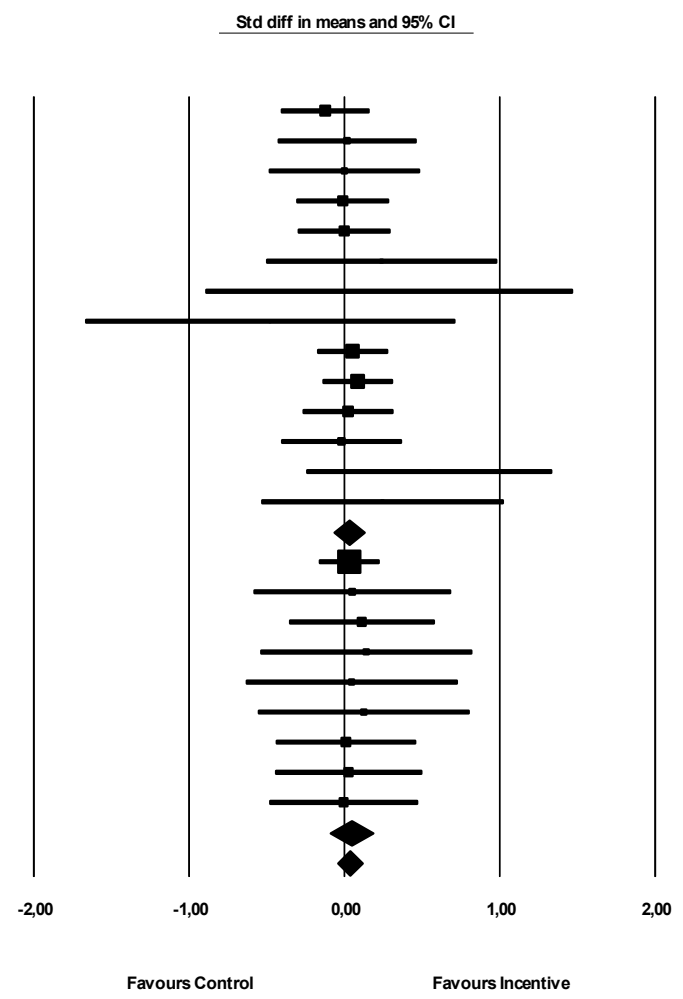

Figure B5. Moderator analysis for passive or interactive BCTs for the post-incentive phase.

Appendix C

Risk of Bias according to the Cochrane risk-of-bias tool

First Author (Year)	RANDOM SEQUENCE GENERATION	ALLOCATION CONCEALMENT	BLINDING OUTCOME ASSIGNEMENTS	INCOMPLETE OUTCOME DATA	SELECTIVE REPORTING	OTHER BIAS
Desai (2020)	+	+	+	-	+	-
Dombrowski (2020)	+	+	+	+	+	+
Faghri (2014)	-	?	?	?	-	+
Finkelstein (2016)	+	+	+	+	+	?
Finkelstein (2017)	-	+	+	+	+	-
Geliebter (2013)	+	+	?	-	?	?
Glanz (2021)	?	+	+	+	+	+
Jeffery (1998)	?	?	?	?	?	+
John (2011)	?	?	-	?	-	+
Kranker (2018)	?	?	?	-	+	?
Kullgren (2013)	+	+	+	+	?	+
Kullgren (2016)	+	+	?	+	?	+
Ladapo (2022)	?	+	+	+	+	+
Leahey (2015)	?	?	+	+	?	+
Memon (2018)	+	+	?	+	?	?
Patel (2016)	+	?	+	+	+	+
Petry (2011)	?	?	?	-	?	?
Pratt (2023)	?	?	+	?	?	+
Pope (2014)	+	+	?	+	?	+
Rounds (2020)	+	+	-	+	?	+
Saccone (1978)	?	?	?	+	?	-
Shin (2017)	+	+	-	+	+	+
Sykes-Muskett (2017)	+	-	-	+	+	+
van der Swaluw (2018)	+	?	-	+	+	+
VanEpps (2019)	?	?	?	+	?	-
Voils (2021)	+	+	?	+	+	+
Volpp (2008)	?	+	-	+	+	?
West (2020)	?	?	+	+	+	+
Wing (1996)	?	?	?	-	?	?

Green: Low risk; Yellow: Unclear; Red: High risk

Appendix D

Funnel Plots

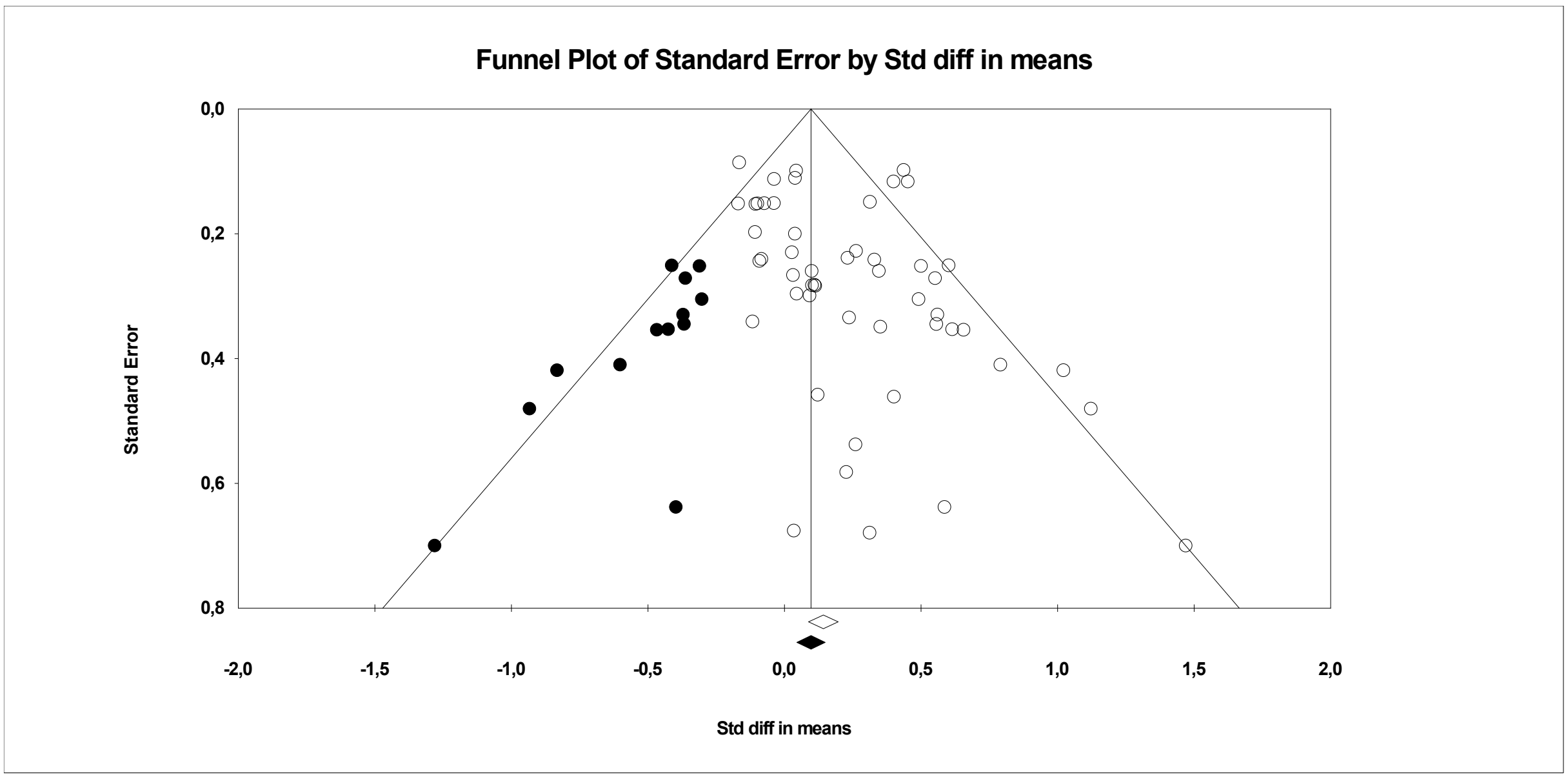

Figure D1. Funnel plot for the incentive phase

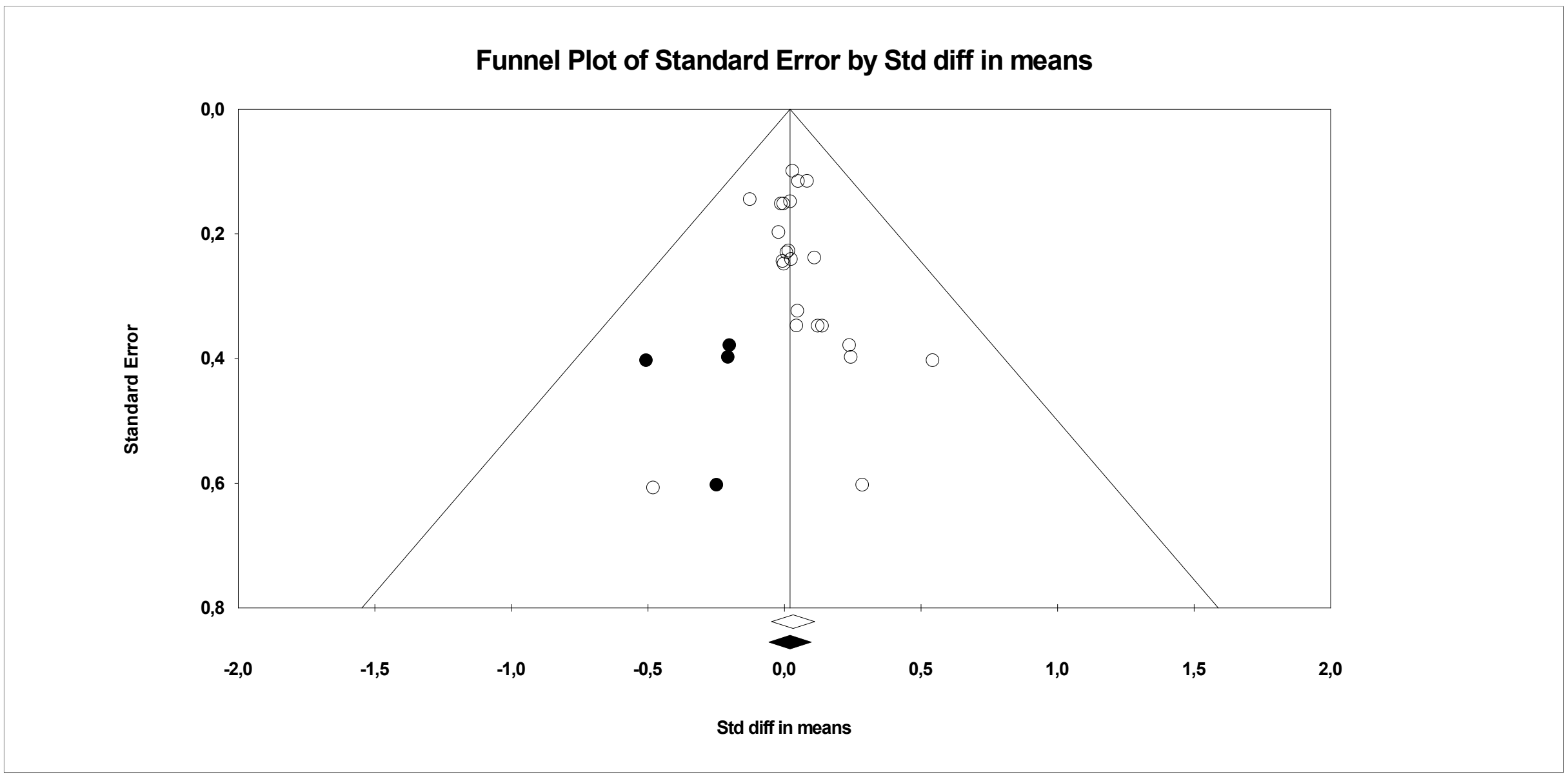

Figure D2. Funnel plot for the post-incentive phase

Supplemental 1 (S1): Detailed search strategies

PUBMED:
((incentiv*[ti] OR financ*[ti] OR "gift card"[ti] OR "gift cards"[ti] OR Loyalty*[ti] OR monetar*[ti] OR money[ti] OR pay[ti] OR payed[ti] OR paying[ti] OR payment*[ti] OR prize*[ti] OR voucher*[ti] OR "cash"[ti] OR discount*[ti] OR lotter*[ti] OR token*[ti] OR raffle*[ti] OR coupon*[ti] OR reimburs*[ti] OR contract[ti] OR contracts[ti] OR contingenc*[ti] OR reward*[ti] OR reinforc*[ti] OR "Reward"[majr] OR "Motivation"[majr:noexp] OR "Intention"[majr] OR "Goals"[majr] OR "Reinforcement, Psychology"[Majr:NoExp] OR "Reward"[majr] OR "Token Economy"[Majr] OR "Extinction, Psychological"[majr] OR "Reinforcement Schedule"[majr] OR "Gift Giving"[majr] OR "Financial Support"[Majr:NoExp] OR "Awards and Prizes"[Majr:NoExp] OR "Reimbursement Mechanisms"[Majr:NoExp] OR "Reimbursement, Incentive"[Majr] OR "Contracts"[Majr] OR "monetary incentive*"[tiab]) AND (eat[ti] OR eating*[ti] OR "Eating"[majr] OR diet*[ti] OR diets[ti] OR "Diet"[majr] OR weight*[ti] OR "Body Weight"[majr] OR overweight[ti] OR "Overweight"[majr] OR waist*[ti] OR "Waist-Hip Ratio"[majr] OR "Waist-Height Ratio"[majr] OR "Waist Circumference"[majr] OR obes*[ti] OR "Obesity"[majr] OR "Feeding Behavior"[majr] OR "Feeding Behavior"[ti] OR "Weight Loss"[majr] OR "Weight Loss"[ti] OR "physical activity"[ti] OR "Exercise"[majr] OR "Exercis*"[ti] OR "Sports"[majr] OR "sport"[ti] OR "sports"[ti] OR "Athletic Performance"[ti] OR "athletics"[ti] OR "Cardiorespiratory Fitness"[ti] OR "Physical Endurance"[ti] OR "Physical Fitness"[ti] OR "Cardiorespiratory Fitness"[ti] OR "Baseball"[ti] OR "Basketball"[ti] OR "Bicycling"[ti] OR "Boxing"[ti] OR "Cricket"[ti] OR "Football"[ti] OR "Golf"[ti] OR "Gymnastics"[ti] OR "Hockey"[ti] OR "Martial Arts"[ti] OR "Tai Ji"[ti] OR "Mountaineering"[ti] OR "Tennis"[ti] OR "Running"[ti] OR "Jogging"[ti] OR "Skating"[ti] OR "Skiing"[ti] OR "Soccer"[ti] OR "Track and Field"[ti] OR "Volleyball"[ti] OR "Walking"[ti] OR "Swimming"[ti] OR "Diving"[ti] OR "Weight Lifting"[ti] OR "Wrestling"[ti] **OR "Body Mass Index"[majr] OR "Body Mass Index"[ti] OR "BMI"[ti]**) AND ("randomized controlled trial"[ptyp] OR "controlled clinical trial"[ptyp] OR "randomized"[tiab] OR "randomised"[tiab] OR "placebo"[tiab] OR "randomly"[tiab] OR "trial"[ti] OR "groups"[ti]) NOT ("Animals"[mesh] NOT "Humans"[mesh]) NOT ((tobacco[ti] OR smoking[ti] OR cigarett*[ti] OR substance abus*[ti] OR alcohol*[ti] OR drinking[ti] OR "Analgesics, Opioid"[majr] OR opioid*[ti] OR opiat*[ti] OR "Substance-Related Disorders"[majr] OR cocain*[ti]) NOT "weight"[ti]) AND (english[la] OR dutch[la]) NOT ("Animals"[mesh] NOT "Humans"[mesh]))

PSYCINFO:
((MM "Monetary Incentives" OR MM "Incentives" OR MM "Monetary Rewards" OR MM "Motivation" OR MM "Achievement Motivation" OR MM "Intrinsic Motivation" OR MM "Extrinsic Motivation" OR MM "Intention" OR MM "Behavioral Intention" OR MM "Goals" OR MM "Reinforcement" OR MM "Differential Reinforcement" OR MM "Positive Reinforcement" OR MM "Reinforcement Schedules" OR MM "Concurrent Reinforcement Schedules" OR MM "Fixed Interval Reinforcement" OR MM "Fixed Ratio Reinforcement" OR MM "Reinforcement Delay" OR MM "Variable Interval Reinforcement" OR MM "Variable Ratio Reinforcement" OR MM "Secondary Reinforcement" OR MM "Contingency Management" OR MM "Token Economy Programs" OR MM "Behavior Contracting" OR TI(incentiv*) OR TI(financ*) OR TI("gift card") OR TI("gift cards") OR TI(Loyalty*) OR TI(monetar*) OR TI(money) OR TI(pay*) OR TI(prize*) OR TI(voucher*) OR TI(cash OR discount*) OR TI(lotter*) OR TI(token*) OR TI(raffle*) OR TI(coupon*) OR TI(reimburs*) OR TI(contract) OR TI(contracts) OR TI(contingenc*) OR TI(reward*) OR TI(reinforc*)) AND (MM "Eating Behavior" OR MM "Food Preparation" OR TI activ* OR TI walk* OR MM "Physical Activity" OR MM "Actigraphy" OR MM "Exercise" OR TI sedentary* OR MM "Sedentary Behavior" OR TI gym* OR TI fitness*[ti] OR MM "Physical Fitness" OR TI exercis* OR TI step* OR TI eat* OR MM "Eating Behavior" OR MM "Binge Eating" OR MM "Dietary Restraint" OR MM "Food Refusal" OR MM "Eating Attitudes" OR TI diet* OR TI diets OR MM "Diets" OR MM "Dietary Restraint" OR MM "Fast Food" OR MM "Food Preparation" OR TI weight* OR MM "Body Weight" OR MM "Birth Weight" OR MM "Overweight" OR MM "Underweight" OR MM "Weight Gain" OR MM "Weight Loss" OR TI overweight[ti] OR TI waist* OR TI obes* OR MM "Obesity" OR TI "travel to school" OR TI "travel to work" OR TI "active travel" OR TI active travel* OR MM "Weight Loss" OR MM "Body Weight" OR MM "Birth Weight" OR MM "Overweight" OR MM "Underweight" OR MM "Weight Control" OR MM "Weight Gain" OR MM "Weight Loss" OR MM "Weight Control" OR MM "Weight Gain" OR DE "Exercise" OR DE "Aerobic Exercise" OR DE "Weightlifting" OR DE "Yoga" OR DE "Sports" OR DE "Athletes" OR DE "Baseball" OR DE "Basketball" OR DE "Extreme Sports" OR DE "Football" OR DE "Judo" OR DE "Martial Arts" OR DE "Professional Sports" OR DE "Soccer" OR DE "Swimming" OR DE "Tennis" OR TI "Weight Loss" OR TI "physical activity" OR TI

Classified as Internal | Intern

"Exercise"[majr] OR TI "Exercis*" OR TI "sport" OR TI "sports" OR TI "Athletic Performance" OR TI "athletics" OR TI "Cardiorespiratory Fitness" OR TI "Physical Endurance" OR TI "Physical Fitness" OR TI "Cardiorespiratory Fitness" OR TI "Baseball" OR TI "Basketball" OR TI "Bicycling" OR TI "Boxing" OR TI "Cricket" OR TI "Football" OR TI "Golf" OR TI "Gymnastics" OR TI "Hockey" OR TI "Martial Arts" OR TI "Tai Ji" OR TI "Mountaineering" OR TI "Tennis" OR TI "Running" OR TI "Jogging" OR TI "Skating" OR TI "Skiing" OR TI "Soccer" OR TI "Track and Field" OR TI "Volleyball" OR TI "Walking" OR TI "Swimming" OR TI "Diving" OR TI "Weight Lifting" OR TI "Wrestling" **OR MM "Body Mass Index" OR DE "Body Mass Index" OR TI "Body Mass Index" OR TI "BMI"**) AND (TX random* OR TI controlled* OR AB controlled* OR SU controlled* OR TX RCT OR TI trial OR SU "Treatment Guidelines") NOT ((TI tobacco OR TI smoking OR TI cigarett* OR TI substance abus* OR TI alcohol* OR TI drinking OR MM "Drug Abuse" OR MM "Alcohol Abuse" OR MM "Drug Dependency" OR MM "Inhalant Abuse" OR MM "Polydrug Abuse" OR MM "Narcotic Drugs" OR MM "Apomorphine" OR MM "Atropine" OR MM "Meperidine" OR MM "Methadone" OR MM "Opiates" OR MM "Tramadol" OR TI opioid* OR TI opiat* OR MM "Drug Usage" OR MM "Alcohol Drinking Patterns" OR MM "Drug Abuse" OR MM "Electronic Cigarettes" OR MM "Intravenous Drug Usage" OR MM "Marijuana Usage" OR MM "Substance Use Disorder" OR MM "Tobacco Smoking" OR TI cocain*) NOT (TI "weight*")) AND (LA english OR LA dutch) NOT (PO "Animal" NOT PU "Human"))

Officieel COCHRANE-RCT-filter voor PsycINFO
(TX random* OR TX control* OR SU "Treatment" OR SU "Adjunctive Treatment" OR SU "Adventure Therapy" OR SU "Aftercare" OR SU "Alternative Medicine" OR SU "Behavior Modification" OR SU "Bibliotherapy" OR SU "Cognitive Techniques" OR SU "Computer Assisted Therapy" OR SU "Creative Arts Therapy" OR SU "Crisis Intervention Services" OR SU "Cross Cultural Treatment" OR SU "Disease Management" OR SU "Health Care Services" OR SU "Hydrotherapy" OR SU "Interdisciplinary Treatment Approach" OR SU "Involuntary Treatment" OR SU "Language Therapy" OR SU "Life Sustaining Treatment" OR SU "Medical Treatment (General)" OR SU "Milieu Therapy" OR SU "Movement Therapy" OR SU "Multimodal Treatment Approach" OR SU "Multisystemic Therapy" OR SU "Online Therapy" OR SU "Outpatient Treatment" OR SU "Pain Management" OR SU "Partial Hospitalization" OR SU "Personal Therapy" OR SU "Physical Treatment Methods" OR SU "Preventive Medicine" OR SU "Psychotherapeutic Techniques" OR SU "Psychotherapy" OR SU "Rehabilitation" OR SU "Relaxation Therapy" OR SU "Sex Therapy" OR SU "Social Casework" OR SU "Sociotherapy" OR SU "Speech Therapy" OR SU "Symptoms Based Treatment" OR SU "Treatment Guidelines")

EMBASE:
((incentiv*.ti OR financ*.ti OR "gift card".ti OR "gift cards".ti OR Loyalty*.ti OR monetar*.ti OR money.ti OR pay*.ti OR prize*.ti OR voucher*.ti OR cash.ti OR discount*.ti OR lotter*.ti OR token*.ti OR raffle*.ti OR coupon*.ti OR reimburs*.ti OR contract.ti OR contracts.ti OR contingenc*.ti OR reward*.ti OR reinforc*.ti OR *"Reward"/ OR *"Motivation"/ OR *"Reinforcement"/ OR *"Gift Giving"/ OR *"Contract"/ OR "monetary incentive*".ti,ab) AND (activ*.ti OR walk*.ti OR gym*.ti OR fitness*.ti OR exp *"Physical Fitness"/ OR exercis*.ti OR exp *"Exercise"/ OR step*.ti OR eat*.ti OR exp *"Eating"/ OR diet*.ti OR diets.ti OR exp *"Diet"/ OR weight*.ti OR exp *"Body Weight"/ OR overweight.ti OR exp *"Overweight"/ OR waist*.ti OR exp *"Waist-Hip Ratio"/ OR exp *"Waist-Height Ratio"/ OR exp *"Waist Circumference"/ OR "travel to school".ti OR "travel to work".ti OR "active travel".ti OR active travel*.ti OR "Feeding Behavior".ti OR exp *"Body Weight Loss"/ OR "Weight Loss".ti OR "physical activity".ti OR exp *"physical activity"/ OR exp *"Exercise"/ OR exp *"physical activity, capacity and performance"/ OR "Exercis*".ti OR exp *"Sport"/ OR "sport".ti OR "sports".ti OR "Athletic Performance".ti OR "athletics".ti OR "Cardiorespiratory Fitness".ti OR "Physical Endurance".ti OR "Physical Fitness".ti OR "Cardiorespiratory Fitness".ti OR "Baseball".ti OR "Basketball".ti OR "Bicycling".ti OR "Boxing".ti OR "Cricket".ti OR "Football".ti OR "Golf".ti OR "Gymnastics".ti OR "Hockey".ti OR "Martial Arts".ti OR "Tai Ji".ti OR "Mountaineering".ti OR "Tennis".ti OR "Running".ti OR "Jogging".ti OR "Skating".ti OR "Skiing".ti OR "Soccer".ti OR "Track and Field".ti OR "Volleyball".ti OR "Walking".ti OR "Swimming".ti OR "Diving".ti OR "Weight Lifting".ti OR "Wrestling".ti **OR *"Body Mass"/ OR "Body Mass Index".ti OR "BMI".ti**) AND (exp "randomized controlled trial"/ OR "crossover procedure"/ OR "double-blind procedure"/ OR "'single-blind procedure"/ OR (random* OR factorial* OR crossover* OR (cross ADJ2 over*) OR placebo* OR (doubl* ADJ2 blind*) OR (singl* ADJ2 blind*) OR assign* OR allocat* OR volunteer*).ti) NOT ((tobacco.ti OR smoking.ti OR cigarett*.ti OR substance abus*.ti OR alcohol*.ti OR drinking.ti OR *"Opiate"/ OR opioid*.ti OR opiat*.ti OR exp *"Drug

Dependence"/ OR cocain*.ti) NOT "weight*".ti) AND (english.la OR dutch.la) AND exp "Humans"/) NOT (conference review or conference abstract).pt

WEB OF SCIENCE:
(ti=(incentiv* OR financ* OR "gift card" OR "gift cards" OR Loyalty* OR monetar* OR money OR pay* OR prize* OR voucher* OR cash OR discount* OR lotter* OR token* OR raffle* OR coupon* OR reimburs* OR contract OR contracts OR contingenc* OR reward* OR reinforc* OR "Reward" OR "Motivation" OR "Reinforcement" OR "Gift Giving" OR "Contract") AND ti=(activ* OR walk* OR gym* OR fitness* OR "Physical Fitness" OR exercis* OR "Exercise" OR step* OR eat* OR "Eating" OR diet* OR diets OR "Diet" OR weight* OR "Body Weight" OR overweight OR "Overweight" OR waist* OR "Waist-Hip Ratio" OR "Waist-Height Ratio" OR "Waist Circumference" OR cholesterol* OR "travel to school" OR "travel to work" OR "active travel" OR active travel* OR "Feeding Behavior" OR "Body Weight Loss" OR "Weight Loss" OR "physical activity" OR "physical activity" OR "Exercise" OR "pphysical activity, capacity and performance" OR "Exercis*" OR "Sport" OR "sport" OR "sports" OR "Athletic Performance" OR "athletics" OR "Cardiorespiratory Fitness" OR "Physical Endurance" OR "Physical Fitness" OR "Cardiorespiratory Fitness" OR "Baseball" OR "Basketball" OR "Bicycling" OR "Boxing" OR "Cricket" OR "Football" OR "Golf" OR "Gymnastics" OR "Hockey" OR "Martial Arts" OR "Tai Ji" OR "Mountaineering" OR "Tennis" OR "Running" OR "Jogging" OR "Skating" OR "Skiing" OR "Soccer" OR "Track and Field" OR "Volleyball" OR "Walking" OR "Swimming" OR "Diving" OR "Weight Lifting" OR "Wrestling" **OR "Body Mass Index" OR "BMI"**) AND **(**TS=("randomized controlled trial" OR "crossover procedure" OR "double-blind procedure" OR "'singleblind procedure") OR ti=(RCT OR random* OR factorial* OR crossover* OR (cross NEAR2 over*) OR placebo* OR (doubl* NEAR2 blind*) OR (singl* NEAR2 blind*) OR assign* OR allocat* OR volunteer*)) NOT TI=((tobacco OR smoking OR cigarett* OR "substance abus*" OR alcohol* OR drinking OR "Opiate" OR opioid* OR opiat* OR "Drug Dependence" OR cocain*) NOT "weight*") AND la=(english OR dutch) NOT ti=(veterinary OR rabbit OR rabbits OR animal OR animals OR mouse OR mice OR rodent OR rodents OR rat OR rats OR pig OR pigs OR porcine OR horse* OR equine OR cow OR cows OR bovine OR goat OR goats OR sheep OR ovine OR canine OR dog OR dogs OR feline OR cat OR cats)) NOT dt=(meeting abstract)

EMCARE:
((incentiv*.ti OR financ*.ti OR "gift card".ti OR "gift cards".ti OR Loyalty*.ti OR monetar*.ti OR money.ti OR pay*.ti OR prize*.ti OR voucher*.ti OR cash.ti OR discount*.ti OR lotter*.ti OR token*.ti OR raffle*.ti OR coupon*.ti OR reimburs*.ti OR contract.ti OR contracts.ti OR contingenc*.ti OR reward*.ti OR reinforc*.ti OR *"Reward"/ OR *"Motivation"/ OR *"Reinforcement"/ OR *"Gift Giving"/ OR *"Contract"/ OR "monetary incentive*".ti,ab) AND (activ*.ti OR walk*.ti OR gym*.ti OR fitness*.ti OR exp *"Physical Fitness"/ OR exercis*.ti OR exp *"Exercise"/ OR step*.ti OR eat*.ti OR exp *"Eating"/ OR diet*.ti OR diets.ti OR exp *"Diet"/ OR weight*.ti OR exp *"Body Weight"/ OR overweight.ti OR exp *"Overweight"/ OR waist*.ti OR exp *"Waist-Hip Ratio"/ OR exp *"Waist-Height Ratio"/ OR exp *"Waist Circumference"/ OR "travel to school".ti OR "travel to work".ti OR "active travel".ti OR active travel*.ti OR "Feeding Behavior".ti OR exp *"Body Weight Loss"/ OR "Weight Loss".ti OR "physical activity".ti OR exp *"physical activity"/ OR exp *"Exercise"/ OR exp *"physical activity, capacity and performance"/ OR "Exercis*".ti OR exp *"Sport"/ OR "sport".ti OR "sports".ti OR "Athletic Performance".ti OR "athletics".ti OR "Cardiorespiratory Fitness".ti OR "Physical Endurance".ti OR "Physical Fitness".ti OR "Cardiorespiratory Fitness".ti OR "Baseball".ti OR "Basketball".ti OR "Bicycling".ti OR "Boxing".ti OR "Cricket".ti OR "Football".ti OR "Golf".ti OR "Gymnastics".ti OR "Hockey".ti OR "Martial Arts".ti OR "Tai Ji".ti OR "Mountaineering".ti OR "Tennis".ti OR "Running".ti OR "Jogging".ti OR "Skating".ti OR "Skiing".ti OR "Soccer".ti OR "Track and Field".ti OR "Volleyball".ti OR "Walking".ti OR "Swimming".ti OR "Diving".ti OR "Weight Lifting".ti OR "Wrestling".ti **OR *"Body Mass"/ OR "Body Mass Index".ti OR "BMI".ti**) AND (exp "randomized controlled trial"/ OR "crossover procedure"/ OR "double-blind procedure"/ OR "'single-blind procedure"/ OR (random* OR factorial* OR crossover* OR (cross ADJ2 over*) OR placebo* OR (doubl* ADJ2 blind*) OR (singl* ADJ2 blind*) OR assign* OR allocat* OR volunteer*).ti) NOT ((tobacco.ti OR smoking.ti OR cigarett*.ti OR substance abus*.ti OR alcohol*.ti OR drinking.ti OR *"Opiate"/ OR opioid*.ti OR opiat*.ti OR exp *"Drug Dependence"/ OR cocain*.ti) NOT "weight*".ti) AND (english.la OR dutch.la) AND exp "Humans"/) NOT (conference review or conference abstract).pt

Classified as Internal | Intern

COCHRANE:
((incentiv* OR financ* OR "gift card" OR "gift cards" OR Loyalty* OR monetar* OR money OR pay* OR prize* OR voucher* OR cash OR discount* OR lotter* OR token* OR raffle* OR coupon* OR reimburs* OR financial contract OR financial contracts OR contingenc* OR reward* OR reinforc* OR "Reward" OR "Motivation" OR "Reinforcement" OR "Gift Giving" OR "Commitment Contract"):ti AND (activ* OR walk* OR gym* OR fitness* OR "Physical Fitness" OR exercis* OR "Exercise" OR step* OR eat* OR "Eating" OR diet* OR diets OR "Diet" OR weight* OR "Body Weight" OR overweight OR "Overweight" OR waist* OR "Waist-Hip Ratio" OR "Waist-Height Ratio" OR "Waist Circumference" OR cholesterol* OR "travel to school" OR "travel to work" OR "active travel" OR active travel* OR "Feeding Behavior" OR "Body Weight Loss" OR "Weight Loss" OR "physical activity" OR "physical activity" OR "Exercise" OR "pphysical activity, capacity and performance" OR "Exercis*" OR "Sport" OR "sport" OR "sports" OR "Athletic Performance" OR "athletics" OR "Cardiorespiratory Fitness" OR "Physical Endurance" OR "Physical Fitness" OR "Cardiorespiratory Fitness" OR "Baseball" OR "Basketball" OR "Bicycling" OR "Boxing" OR "Cricket" OR "Football" OR "Golf" OR "Gymnastics" OR "Hockey" OR "Martial Arts" OR "Tai Ji" OR "Mountaineering" OR "Tennis" OR "Running" OR "Jogging" OR "Skating" OR "Skiing" OR "Soccer" OR "Track and Field" OR "Volleyball" OR "Walking" OR "Swimming" OR "Diving" OR "Weight Lifting" OR "Wrestling" **OR "Body Mass Index" OR "BMI"):**ti NOT ((tobacco OR smoking OR cigarette OR "substance abuse" OR alcohol OR drinking OR "Opiate" OR opioids OR opiate OR opiates OR "Drug Dependence" OR cocaine) NOT "weight"):ti)

NOT (conference abstract:pt)

ACADEMIC SEARCH PREMIER:
(ti(incentiv* OR financ* OR "gift card" OR "gift cards" OR Loyalty* OR monetar* OR money OR pay* OR prize* OR voucher* OR cash OR discount* OR lotter* OR token* OR raffle* OR coupon* OR reimburs* OR contract OR contracts OR contingenc* OR reward* OR "Reward" OR "Motivation" OR "Reinforcement" OR "Gift Giving" OR "Contract") AND ti(activ* OR walk* OR gym* OR fitness* OR "Physical Fitness" OR exercis* OR "Exercise" OR step* OR eat* OR "Eating" OR diet* OR diets OR "Diet" OR weight* OR "Body Weight" OR overweight OR "Overweight" OR waist* OR "Waist-Hip Ratio" OR "Waist-Height Ratio" OR "Waist Circumference" OR cholesterol* OR "travel to school" OR "travel to work" OR "active travel" OR active travel* OR "Feeding Behavior" OR "Body Weight Loss" OR "Weight Loss" OR "physical activity" OR "physical activity" OR "Exercise" OR "pphysical activity, capacity and performance" OR "Exercis*" OR "Sport" OR "sport" OR "sports" OR "Athletic Performance" OR "athletics" OR "Cardiorespiratory Fitness" OR "Physical Endurance" OR "Physical Fitness" OR "Cardiorespiratory Fitness" OR "Baseball" OR "Basketball" OR "Bicycling" OR "Boxing" OR "Cricket" OR "Football" OR "Golf" OR "Gymnastics" OR "Hockey" OR "Martial Arts" OR "Tai Ji" OR "Mountaineering" OR "Tennis" OR "Running" OR "Jogging" OR "Skating" OR "Skiing" OR "Soccer" OR "Track and Field" OR "Volleyball" OR "Walking" OR "Swimming" OR "Diving" OR "Weight Lifting" OR "Wrestling" **OR "Body Mass Index" OR "BMI"**)) AND TI("randomized controlled trial" OR "crossover procedure" OR "double-blind procedure" OR "'single-blind procedure" OR random* OR factorial* OR crossover* OR assign* OR allocat* OR volunteer*) NOT (ti(tobacco OR smoking OR cigarett* OR "substance abus*" OR alcohol* OR drinking OR "Opiate" OR opioid* OR opiat* OR "Drug Dependence" OR cocain*) NOT "weight*") NOT ti(veterinary OR rabbit OR rabbits OR animal OR animals OR mouse OR mice OR rodent OR rodents OR rat OR rats OR pig OR pigs OR porcine OR horse* OR equine OR cow OR cows OR bovine OR goat OR goats OR sheep OR ovine OR canine OR dog OR dogs OR feline OR cat OR cats))

File S2 Standardized, pre-piloted manual for screening and data extraction

Screening

Screening phase 1: Title and Abstract (ASReview & Rayyan)

When a study does not seem to meet the eligibility criteria (see '1b ELIGIBILITY' of the manual) based on the information provided in the title and abstract, the study is marked in ASReview and Rayyan as 'exclude / irrelevant'. Alternatively, the study is marked as 'include / relevant'.

Screening phase 2: Full-text (Excel file)

The full text of the studies that are marked as 'include / relevant' will be consulted to further check if these studies meet the eligibility criteria (using '1b ELIGIBILITY' of the manual). A coding scheme (in Excel) corresponding the eligibility criteria in the below screening manual is used to note down the screening decisions. Reasons for exclusion will also be noted down.

Screening manual

	Variable	Explanation	Categories
**1a. GENERAL INFORMATION**			
**A**	REF	Reference citation in APA (7th)	
**B**	STUDY_ID	Study ID (surname first author and year published e.g., Smith 2001)	
**C**	DATE	Date form completed	
**D**	NAME	Name/ID of person extracting data	
**E**	AUTHOR	Study author contact details (correspondence)	
**1b. ELIGIBILITY**			
**F**	LANG	Language: originally written in English	No<br>Yes
**G**	COM	Completeness: the full text of the article is present and available	No<br>Yes
**H**	RCT	Type of study: randomized controlled trial or quasi-randomized controlled trial (when quasi-RCT, e.g. cluster randomized controlled trial, explain at Notes eligibility)	No<br>Yes<br>Missing
**I**	PARTIC	Participants: adult population (mean age sample ≥18 yrs.)	No<br>Yes<br>Missing
**J**	INCENTIVES		No<br>Yes

		Type of intervention: Intervention consisting of material incentives (cash, vouchers, goods or services, lottery)	Missing
**K**	WEIGHTLOSS	Type of intervention: Intervention aims to (indirectly) promote weight loss (for example by focusing on PA, gym attendance, eating behavior, or weight loss)	No<br>Yes<br>Missing
**L**	COMPARISON	Type of comparison: Control groups with no incentives, so that the effect of the incentives can be isolated	No<br>Yes<br>Missing
**M**	OUTC_WEIGHT	Intervention reports on weight (loss) (kg, lbs. or BMI) at least before and after the intervention.	No<br>Yes<br>Missing
**N**	RECIPIENT	Recipient of incentive is an individual	No<br>Yes<br>Missing
**O**	EXCL_CRIT	Double check these exclusion criteria for <u>exclusion</u>:	None<br>Incentives not contingent on behavioral or outcome achievement (e.g., non-contingent payment, like solely receiving money for attendance at research measurement appointments)<br>Incentives indirectly targeted at individual via groups/ healthcare/practitioner / partner / parent<br>Incentives are not for participant, but for charity.<br>The study is only focused on weight maintenance and not weight loss.<br>Secondary analysis of data that is already used in a previous paper<br>Missing
**P**	INCL_EXCL	Study included or excluded?	Exclude<br>Include<br>Doubt due to missing information

**Q**	NOTES ELIGIBILITY	Note down any remarks or extra explanation (e.g., when the author needs to be contacted for further details and for which details, or give an extra explanation for the reason for exclusion).	…

Screening phase 3: Reference screening

Additional studies will be identified by searching reference lists of relevant reviews, protocol studies and trial registrations, and eligible articles found in phase 1 and 2.
